# *OsBph32* Contributes to Coordinated Cell Wall and Metabolic Responses in Rice Resistance to Brown Planthopper

**DOI:** 10.3390/plants15142132

**Published:** 2026-07-10

**Authors:** Lulu Wang, Ting Liang, Aoyun Zhu, Juansheng Ren, Fangyuan Gao, Guangjun Ren, Renshan Zhu, Xianting Wu

**Affiliations:** 1 College of Life Sciences, Wuhan University, Wuhan 430072, China; 2 State Key Laboratory of Hybrid Rice, Wuhan University, Wuhan 430072, China; 3 College of Electronics and Information Engineering, Shenzhen University, Shenzhen 518060, China; 4 Sichuan Academy of Agricultural Sciences, Chengdu 610066, China

**Keywords:** *OsBph32*, brown planthopper resistance, multi-omics, phenylpropanoid metabolism, cell wall, photosynthetic carbon metabolism

## Abstract

The brown planthopper (BPH, *Nilaparvata lugens*) is a major insect pest of rice (*Oryza sativa* L.) causing severe yield losses across Asia. Although the resistance gene *OsBph32* from the cultivar Ptb33 enhances BPH resistance, its molecular and physiological mechanisms remain unclear. Here, we investigated its function using *OsBph32*-overexpressing lines combined with physiological, transcriptomic, and metabolomic analyses. Overexpression of *OsBph32* in the susceptible cultivar 9311 significantly increased resistance to BPH, as indicated by reduced plant damage and suppressed insect growth. This was associated with increased reactive oxygen species accumulation and callose deposition, suggesting activation of early defense responses. Multi-omics analyses revealed that *OsBph32* is associated with transcriptional changes in genes involved in cell wall biosynthesis, phenylpropanoid metabolism, and carbon metabolism. Metabolomic profiling further showed increased accumulation of flavonoids, phenolamides, and lignin-related metabolites under BPH infestation, together with changes in carbon metabolism and starch accumulation. Collectively, these results suggest that *OsBph32* is associated with coordinated changes in structural reinforcement, secondary metabolism, and carbon metabolism during insect attack, which may contribute to enhanced rice resistance and provide new insights into non-NLR-mediated insect defense mechanisms in plants.

## 1. Introduction

Rice is a staple food crop worldwide, and its production is severely threatened by the brown planthopper (BPH, *Nilaparvata lugens*). BPH is a devastating pest that can cause yield losses of up to 52% without intervention [1,2]. BPH feeds on rice phloem sap, resulting in severe water and nutrient loss in plants. Simultaneously, the callose deposition induced by its feeding blocks the vascular bundles, hindering nutrient transport and accelerating plant wilting, which is a critical factor leading to significant yield reduction or even complete crop failure [3]. To control BPH infestations, various chemical pesticides are widely used. However, their overuse can trigger resistance resurgence in BPH biotypes and also poses significant environmental risks [4]. Therefore, identifying BPH resistance genes from diverse germplasm and developing resistant rice varieties is considered one of the most effective, economical, and environmentally friendly approaches for BPH management [5].

To date, over 40 BPH resistance genes have been identified from cultivated and wild rice varieties, predominantly clustered on chromosomes 3, 4, 6, 8, and 12 [6,7]. Molecular cloning has revealed diverse functional mechanisms: *Bph14* and *Bph26* encode CC-NB-LRR proteins mediating effector-triggered immunity [8,9]; *Bph3* encodes plasma membrane-localized lectin receptor kinases conferring broad-spectrum resistance [10]; and *Bph29* and *Bph30* function through transcriptional regulation and cell wall reinforcement, respectively [11,12]. The resistance gene *OsBph32*, mapped to the short arm of chromosome 6 in Ptb33, encodes a unique short consensus repeat (SCR) domain protein [13]. Introduction of *OsBph32* into the susceptible variety Kasalath enhances BPH resistance, with expression induced in leaf sheaths—the primary feeding site—upon infestation [13]. Unlike the well-characterized mechanisms of the aforementioned genes, however, the molecular basis of *OsBph32*-mediated resistance remains to be elucidated, warranting further investigation to fully harness its potential in breeding programs.

Through long-term evolutionary conflict and co-adaptation, rice has developed a sophisticated defense system in its arms race with the BPH [14]. At the structural level, vascular bundle reinforcement constitutes the first critical barrier against insect attack. Studies have shown that resistance genes such as *Bph6*, *Bph30*, and *Bph40* confer resistance through callose deposition-mediated sieve tube occlusion, as well as coordinated accumulation of lignin, cellulose, and hemicellulose that strengthens cell walls, thereby forming a physical barrier that impedes BPH stylet penetration and sustained feeding [12,15]. At the chemical defense level, rice deploys both constitutive and induced metabolites: the former, including defensive proteins, gramine, and phenolamides, can directly inhibit feeding or exert toxicity [16,17,18]; the latter are activated upon BPH infestation through metabolic reprogramming, notably with jasmonic acid signaling and flavonoid biosynthesis pathways being prominently induced, leading to accumulation of insect-resistant flavonoids such as eriodictyol, naringenin, and quercetin [19,20]. The synergistic action of these multilayered defense mechanisms collectively constitutes the complex network by which rice defends against BPH [21].

The development of omics technologies has provided powerful tools for dissecting plant-insect interactions at the molecular level. Recent molecular and integrated omics studies have revealed that rice defense against BPH involves the activation of phenylpropanoid, flavonoid, and jasmonic acid pathways, along with the accumulation of related metabolites [22,23,24]. The MYC2-JAMYB transcriptional cascade further coordinates metabolic reprogramming across phenylpropanoid, phenolamide, and volatile biosynthesis pathways to regulate direct and indirect defenses [25]. Although callose deposition and cell wall reinforcement have been reported in BPH resistance, the coordination between physical defense and metabolic responses remains to be further clarified.

In this study, we generated *OsBph32*-overexpressing lines and investigated their responses to BPH infestation using integrated multi-omics and physiological analyses. Our results suggest that *OsBph32* is associated with multiple defense-related processes, including cell wall-related structural changes, changes in phenylpropanoid-derived metabolites, and alterations in photosynthetic carbon metabolism under BPH infestation. Integrated analysis further suggests changes in phenylpropanoid pathway metabolism, with coordinated shifts in lignin and flavonoid biosynthesis. These findings suggest that *OsBph32* may be associated with coordinated physical, chemical, and metabolic responses, providing new insights into rice–BPH interactions and suggesting that *OsBph32* may be a useful target for breeding insect-resistant rice.

## 2. Results

### 2.1. OsBph32 Overexpression Enhanced Rice Resistance to BPH Infestation

**Figure 1:**
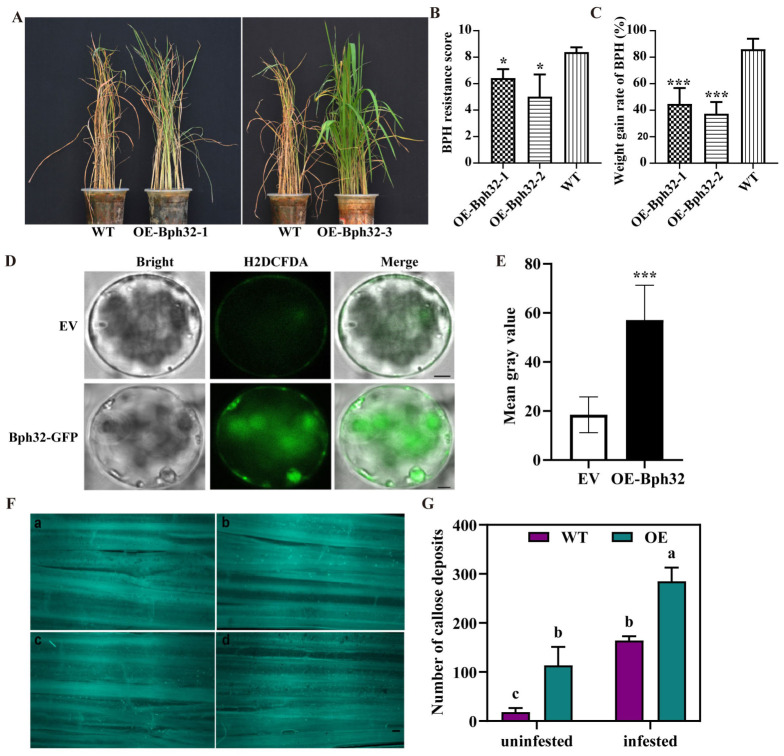
*OsBph32* overexpression enhanced rice resistance to BPH. (**A**): BPH resistance phenotypes of OE-*Bph32* transgenic plants at the seedling stage. (**B**): BPH resistance scores of OE-*Bph32* and WT plants at the seedling stage. (**C**): Resistance identification based on the BPH weight gain method. (**D**): ROS accumulation in rice protoplasts transiently expressing OsBph32-GFP detected by H_2_DCFDA staining (Scale bar = 5 µm). (**E**): Quantification of relative fluorescence intensity in rice protoplasts. Fluorescence intensity was quantified from 15 independent protoplasts for each treatment using ImageJ (version 1.54g). (**F**): Fluorescence microscope observation of callose deposition in leaf sheaths of WT (**a**) and OE-*Bph32* plants (**b**) without BPH infestation and WT (**c**) and OE-*Bph32* plants (**d**) after BPH infestation for 24 h. Scale bar = 200 µm. (**G**): Quantification of callose deposits in leaf sheaths using ImageJ (version 1.54g). Statistical significance in (**B**,**C**,**E**) was analyzed by Student’s *t*-test. Statistical significance in (**G**) was analyzed by two-way ANOVA followed by Tukey’s multiple comparisons test. Different letters indicate significant differences among groups. Asterisks indicate significant differences between OE-*Bph32* and WT plants: *, *p* < 0.05; ***, *p* < 0.001.

Previous studies have reported that introducing the *OsBph32* cDNA from rice variety Ptb33 into the susceptible indica variety Kasalath significantly enhanced its resistance to BPH [13]. To elucidate the role of *OsBph32* in rice defense against BPH, we constructed an *OsBph32* overexpression vector driven by the CaMV35S promoter and transformed it into the susceptible indica variety 9311 (Figure S1A). Three independent T2 generation lines were selected and validated for *OsBph32* expression by qRT-PCR, and OsBph32-GFP protein accumulation was further confirmed by Western blot analysis using an anti-GFP antibody (Figure S1B,C). Therefore, OE-*Bph32*-1 and OE-*Bph32*-3 were selected for subsequent BPH resistance assays and physiological analyses. BPH resistance evaluation showed that the seedling damage scores of OE-*Bph32*-1 and OE-*Bph32*-3 were 6.417 ± 0.392 and 5.017 ± 0.971, respectively, both of which were significantly lower than that of WT plants (8.383 ± 0.209). In addition, the BPH weight gain rates on OE-*Bph32*-1 and OE-*Bph32*-3 plants were 44.795 ± 4.878% and 37.380 ± 3.940%, respectively, which were significantly lower than that on WT plants (86.008 ± 3.542%) (Figure 1A–C). These results indicate that *OsBph32* overexpression lines exhibited enhanced resistance to BPH.

Given that ROS are key signaling molecules in plant defense responses [26], we examined the effect of *OsBph32* on ROS dynamics. The results showed that rice protoplasts transiently expressing OsBph32-GFP exhibited enhanced ROS accumulation compared with the control (Figure 1D,E). Upon BPH infestation, OE-*Bph32* plants showed elevated H_2_O_2_ levels, reaching approximately two-fold that of WT at 48 h, along with increased POD and CAT activities at 12 h and an approximately 1.5-fold increase in SOD activity at 24 h (Figure S1D,E). Callose deposition is an important physical barrier that prevents BPH from feeding on phloem sap in rice [27]. Further analysis revealed that *OsBph32* overexpression significantly promoted callose deposition (Figure 1F). Although the number of callose deposits in leaf sheaths increased after BPH infestation in both genotypes, the increase was greater in OE-*Bph32* plants than in WT plants (Figure 1G). After BPH infestation, the activity of the callose-hydrolyzing enzyme β-1,3-glucanase was generally lower in OE-*Bph32* plants than in WT plants, with significant differences observed at several time points (Figure S1G). In addition, the expression levels of callose-related GNS family genes were lower in OE-*Bph32* plants after BPH infestation (Figure S1F). Together, these results suggest that *OsBph32* overexpression enhances rice resistance to BPH, accompanied by increased ROS accumulation and callose deposition.

### 2.2. OsBph32 Overexpression Amplified Transcriptional Reprogramming upon BPH Infestation

To further investigate the transcriptional responses associated with *OsBph32*-mediated insect resistance in rice, leaf sheaths were selected from WT plants un-infested with BPH (U-WT), WT plants infested with BPH for 24 h (I-WT), OE-*Bph32* plants un-infested with BPH (U-OE) and OE-*Bph32* plants infested with BPH for 24 h (I-OE) for transcriptome sequencing analysis. Differentially expressed genes (DEGs) were screened using |log_2_FC| ≥ 1 and FDR ≤ 0.01 as thresholds.

Under uninfested conditions, 1723 DEGs (958 up, 765 down) were detected in OE-*Bph32* compared with WT. After 24 h of BPH infestation, a total of 2762 DEGs (1515 up, 1247 down) were shown in OE-*Bph32* compared with WT plants. Notably, BPH infestation triggered 6296 DEGs in OE-*Bph32* plants (3523 up, 2773 down), compared with 3854 DEGs in WT plants (2469 up, 1385 down) (Table S2 and Figure S2). These results indicated that BPH infestation broadly alters gene expression, and *OsBph32* overexpression was associated with a larger transcriptional response after BPH infestation.

### 2.3. Functional Annotation and Pathway Analysis of DEGs in OE-Bph32 Plants

**Figure 2:**
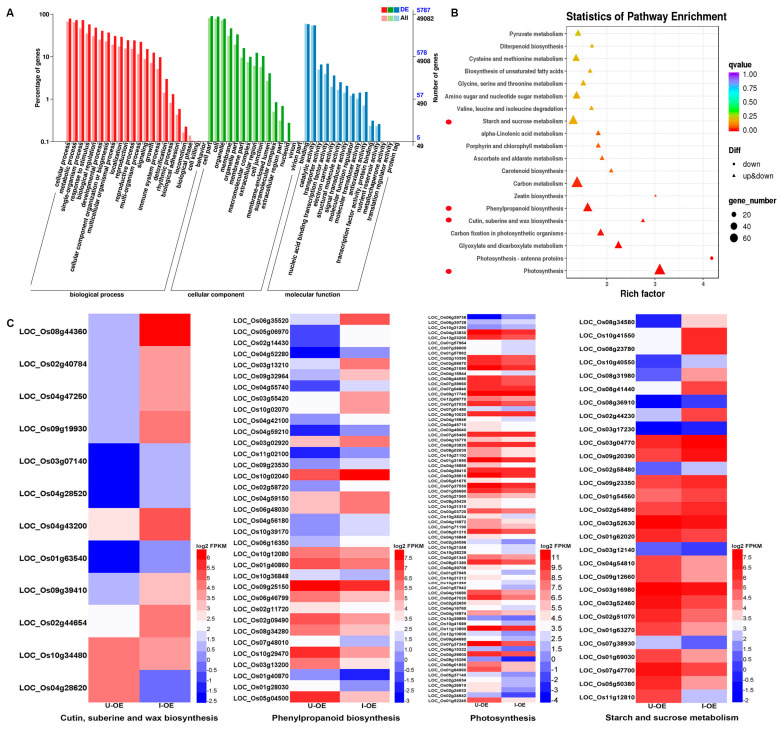
GO annotation and KEGG pathway analysis of DEGs in OE-*Bph32* before and after BPH infestation. (**A**): GO term annotation. (**B**): KEGG pathway enrichment analysis. (**C**): Heat map of DEGs in selected metabolic pathways.

GO enrichment analysis was performed on the DEGs in OE-*Bph32* plants with and without 24 h BPH feeding (U-OE vs. I-OE). In biological processes, DEGs were mainly enriched in response to stimulus, immune system processes, detoxification, and multicellular organism processes. In cellular components, DEGs were mainly enriched in the cell membrane, extracellular region, and cell junction. In terms of molecular functions, DEGs were mainly enriched in transcription factor activity, antioxidant activity, and electron carrier activity (Figure 2A). KEGG pathway analysis showed that DEGs were concentrated in the cutin, suberization and wax biosynthesis, phenylpropanoid biosynthesis, photosynthetic metabolism, and starch and sucrose metabolism (Figure 2B,C). The photosynthetic metabolism pathway was found to be the most significant, with 76 DEGs, of which 73 DEGs were down-regulated. Additionally, the ascorbate and aldarate metabolism pathway, linked to ROS burst and scavenging, was also prominently enriched (Figure 2C). MapMan analysis further displayed the distribution of DEGs across biological processes in OE-*Bph32* plants before and after BPH feeding (Figure S3).

GO enrichment analysis of DEGs between BPH-infested OE-*Bph32* and WT plants (I-OE vs. I-WT) revealed enrichment in biological processes related to stimulus response, immune system processes, and detoxification; cellular components enriched in the extracellular region; and molecular functions including transcription factor activity, antioxidant activity, and signal transduction (Figure S4A). KEGG pathway analysis showed significant enrichment in diterpenoid antitoxin, phenylpropanoid biosynthesis, and plant hormone signal transduction pathways (Figure S4B,C). MapMan analysis further identified differentially expressed genes involved in transcription factors, protein modification/degradation, hormone signaling, ROS scavenging (e.g., peroxisome, glutathione S-transferase), and primary/secondary metabolism including cell wall synthesis, phenylpropanoids, and terpenoids (Figure S5).

To validate the reliability of the RNA-seq data, 10 representative DEGs were selected from each of the two comparisons, I-WT vs. I-OE and U-OE vs. I-OE, resulting in a total of 20 DEGs for qRT-PCR analysis. Linear regression analysis between qRT-PCR and RNA-seq log_2_ fold-change values showed high consistency between the two methods, with R^2^ values of 0.9252 for I-WT vs. I-OE and 0.8960 for U-OE vs. I-OE (Figure S6). These results support the reliability of the RNA-seq data.

### 2.4. OsBph32 Is Associated with Reprogramming of Photosynthetic Carbon Metabolism and Starch Accumulation During BPH Infestation

**Figure 3:**
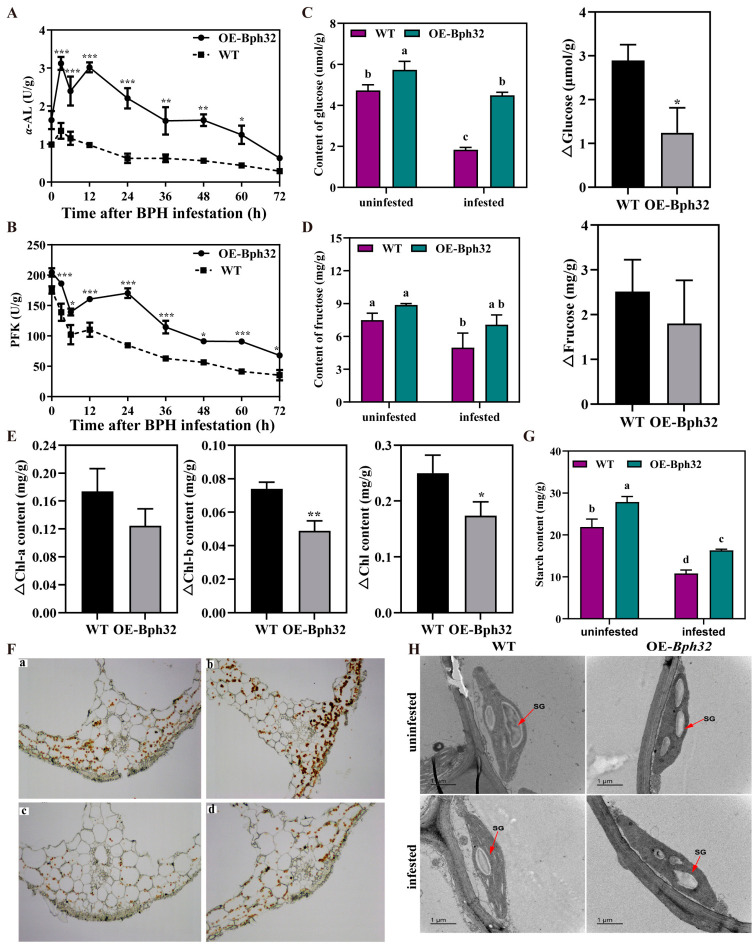
*OsBph32* overexpression is associated with altered photosynthetic carbon metabolism and starch accumulation during BPH resistance. (**A**): Changes in α-AL activity after BPH feeding. (**B**): Changes in PFK activity after BPH feeding. The time points in A and B were 0, 3, 6, 12, 24, 36, 48, 60, and 72 h after BPH infestation. (**C**): Glucose content under uninfested and infested conditions and Δglucose after BPH feeding. (**D**): Fructose content under uninfested and infested conditions and Δfructose after BPH feeding. (**E**): Changes in chlorophyll content (chlorophyll a, chlorophyll b and total chlorophyll) after BPH feeding. (**F**): Cytological observation of starch granules in transverse sections of leaf sheaths. (**a**), WT without BPH feeding; (**b**), OE-*Bph32* without BPH feeding; (**c**), WT with BPH feeding; (**d**), OE-*Bph32* with BPH feeding. Scale bar = 100 µm. (**G**): Starch content in rice leaf sheaths. (**H**): Observation of starch granules in chloroplasts by transmission electron microscopy. Scale bar = 1 µm. Statistical significance was analyzed by two-way ANOVA followed by Sidak’s multiple comparisons test for (**A**,**B**), two-way ANOVA followed by Tukey’s multiple comparisons test for (**C**,**D**,**G**), and Student’s *t*-test for the Δ values in (**C**–**E**). Different letters indicate significant differences among groups. Asterisks indicate significant differences between OE-*Bph32* and WT plants. * *p* < 0.05; ** *p* < 0.01; *** *p* < 0.001.

Transcriptomic data suggested that photosynthetic pathways were associated with the response to BPH infestation and that phloem feeding may affect carbon metabolism. Therefore, we measured the activities of α-amylase (α-AL) and phosphofructokinase (PFK) in rice leaf sheaths at 0–72 h after infestation. After BPH feeding, α-AL activity initially increased then decreased in both OE-*Bph32* and WT plants, but the increase was more pronounced in *OE-Bph32* plants (Figure 3A). PFK activity declined overall, but the decrease was slower and levels remained higher in OE-*Bph32* plants at most post-infestation time points (Figure 3B). Under normal conditions, glucose content was higher in OE-*Bph32* plants than in WT, whereas fructose content showed no significant difference between the two genotypes. After 24 h of BPH feeding, both glucose and fructose contents decreased, but the reduction in glucose content was smaller in OE-*Bph32* plants than in WT (Figure 3C,D), suggesting that *OsBph32* overexpression may help maintain soluble sugar levels during BPH feeding. Chlorophyll analysis showed that OE-*Bph32* plants had significantly smaller decreases in chlorophyll b and total chlorophyll than WT (Figure 3E), indicating that *OsBph32* overexpression may help maintain chlorophyll content under BPH feeding.

As the major energy reserve, starch accumulation reflects plant energy status [28]. Histochemical staining, starch content measurements, and transmission electron microscopy showed that starch granule accumulation in OE-*Bph32* leaf sheaths was higher than in wild-type regardless of infestation (Figure 3F–H), suggesting a shift toward starch accumulation or carbon storage in OE-*Bph32* plants. Additionally, OE-*Bph32* significantly reduced grain chalkiness and amylose content, with endosperm microstructure showing more uniform and compact starch granules (Figure S7), indicating that *OsBph32* may contribute to improved grain quality by affecting starch granule organization in the endosperm.

### 2.5. OsBph32 Enhanced BPH Resistance Is Associated with Cell Wall Remodeling and Lignin Deposition

**Figure 4:**
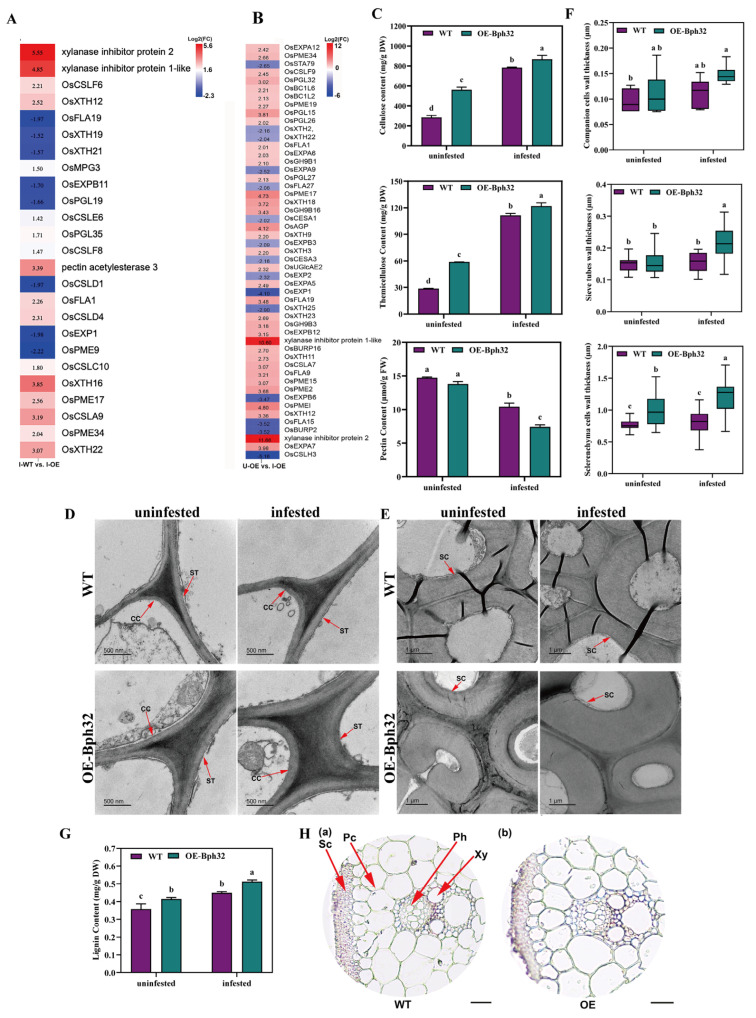
*OsBph32* overexpression is associated with cell wall remodeling and lignin deposition. (**A**): The gene expression profile of OE-*Bph32* and WT plants after BPH feeding. (**B**): The gene expression profile of OE-*Bph32* plants before and after BPH feeding. (**C**): Contents of cell wall components (cellulose, hemicellulose and pectin) in OE-*Bph32* and WT plants before and after BPH infestation. (**D**): The cell wall thickness of companion cells and sieve tubes in the phloem of leaf sheath observed by transmission electron microscopy. CC: companion cell; ST: sieve tube. Scale bars = 500 nm. (**E**): Cell wall thickness of sclerenchyma cells in leaf sheaths observed by transmission electron microscopy. Scale bars = 1 µm. (**F**): Quantification of cell wall thickness in companion cells, sieve tubes and sclerenchyma cells. (**G**): Lignin accumulation by spectrophotometry. (**H**): Histochemical staining of lignin in WT plants (**a**) and OE-*Bph32* plants (**b**) after 24 h of BPH infestation. Sc, sclerenchyma cell; Pc, parenchymal cells; Xy, xylem; Ph, phloem. Scale bars = 50 µm. Statistical significance in (**C**,**F**,**G**) was analyzed by two-way ANOVA followed by Tukey’s multiple comparisons test. Different letters indicate significant differences among groups at *p* < 0.05.

As the structural foundation of plants, the plant cell wall serves as the primary physical barrier against pests and diseases. Its main components include polysaccharides such as cellulose, hemicellulose, and pectin, as well as secondary metabolites like lignin, cutin, and wax [29,30]. Transcriptome analysis revealed differential expression of multiple genes related to cell wall remodeling. Compared with WT plants, OE-*Bph32* plants exhibited significantly upregulated expression of genes associated with pectin metabolism (e.g., *OsPME17*), hemicellulose synthesis (e.g., *OsXTH16*), and xylanase inhibitor protein-encoding genes after 24 h of BPH feeding (I-WT vs. I-OE) (Figure 4A). Further analysis indicated that, in response to BPH feeding in OE-*Bph32* plants (U-OE vs. I-OE), genes involved in cellulose synthesis, pectin metabolism, and hemicellulose synthesis (involving multiple members of the *XTH* and *CSL* gene families) were also significantly induced (Figure 4B).

Further changes in the cell wall main components (cellulose, hemicellulose, pectin) were detected. The results showed that the contents of cellulose and hemicellulose in OE-*Bph32* plants were significantly higher than those in WT plants both before and after BPH feeding. Conversely, pectin content decreased after feeding, with a greater reduction observed in OE-*Bph32* plants compared to the WT (Figure 4C). Transmission electron microscopy revealed changes in the cell wall thickness of companion cells, sieve tubes, and sclerenchyma cells in leaf sheaths after BPH feeding, with significant increases in OE-*Bph32* plants being more evident in sieve tubes and sclerenchyma cells (Figure 4D–F). Furthermore, as the primary component of secondary cell walls, lignin was also detected. Quantitative analysis showed that under normal conditions, lignin content in OE-*Bph32* plants was significantly higher than that in WT plants. After 24 h of BPH feeding, lignin content significantly increased in OE-*Bph32* plants and remained higher than that in the WT plants (Figure 4G). Histochemical staining further confirmed that after 24 h of feeding, the parenchymal cells and xylem cells in OE-*Bph32* plants exhibited deeper staining, indicating higher lignin accumulation levels compared to the WT plants (Figure 4H).

### 2.6. Metabolome Data Evaluation and Differential Abundance Metabolite Summary

To investigate metabolic changes associated with *OsBph32*-mediated resistance to the BPH, we performed metabolomic analysis using the same samples (U-OE, U-WT, I-OE, and I-WT) as those used for transcriptome sequencing. A total of 904 metabolites were detected. Differential abundance metabolites (DAMs) were screened using the OPLS-DA model with VIP ≥ 1 combined with fold change criteria (fold change ≥ 2 or ≤ 0.5). The results showed that before BPH feeding, 48 DAMs were identified between OE-*Bph32* and WT plants (25 upregulated, 23 downregulated). After 24 h of BPH feeding, the number of DAMs between the two genotypes increased to 174 (38 upregulated, 136 downregulated). In addition, 104 DAMs were identified in OE-*Bph32* plants before versus after BPH feeding (46 upregulated, 58 downregulated) (Figure S8A,B). Principal component analysis revealed clear separation of metabolic profiles among the four sample groups (Figure S8C), and the high correlation between biological replicates (r^2^ > 0.95) indicated the reliability of the data (Figure S8D). Cluster analysis showed that flavonoids, lipids, and phenolics were the most abundant classes of metabolites accumulated across samples (Figure S8E).

### 2.7. Metabolite Clustering and Pathway Enrichment in OE-Bph32 Plants Under BPH Infestation

**Figure 5:**
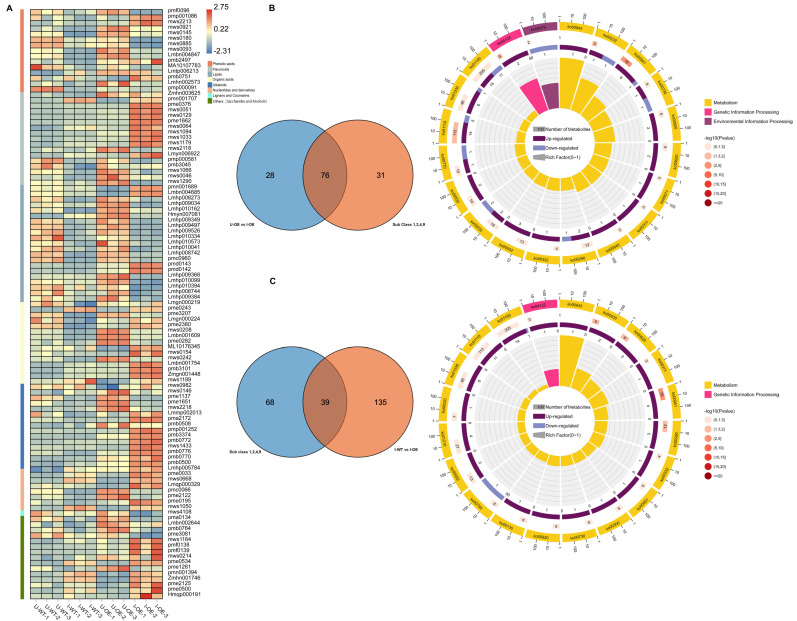
Metabolite clustering and KEGG enrichment analysis of DAMs. (**A**): Cluster analysis of metabolites in Subclasses 1, 2, 4 and 9. (**B**): KEGG enrichment analysis of four classes of DAMs in U-OE vs. I-OE. (**C**): KEGG enrichment analysis of four classes of DAMs in I-WT vs. I-OE.

To assess metabolite trends across sample groups, we performed z-score normalization and k-means clustering on mean relative abundances. Metabolites clustered into nine subclasses, with subclasses 1, 2, 4, and 9 (total 107 metabolites) showing increased levels upon *OsBph32* overexpression or BPH infestation (Figure S9). Heatmap analysis revealed these were primarily flavonoids, phenolamides, alkaloids, lipids, and sugars, suggesting that these metabolite classes were associated with the response to BPH infestation (Figure 5A). Further analysis revealed that compared to U-OE, the quillaic acid showed the highest fold change (FC = 11.39) in I-OE, while flavonoids such as kaempferol, acacetin, and apigenin were also highly accumulated (Table S3). KEGG enrichment analysis indicated that the 76 DAMs between U-OE and I-OE were primarily enriched in secondary metabolite synthesis (ko01110) and flavonoid biosynthesis pathways (ko00941) (Figure 5B). Compared with I-WT, the I-OE group showed the greatest upregulation of the flavonoid genkwanin (FC = 13.58), followed by D-galactose (FC = 12.50), D-mannose (FC = 7.26), and the phenolamide compound N-p-coumaroyltyramine (FC = 11.75) (Table S3). KEGG analysis revealed that 39 DAMs in I-OE vs. I-WT were significantly enriched in flavonoid biosynthesis (ko00941) and valine, leucine, and isoleucine biosynthesis pathways (ko00290) (Figure 5C). These results suggest that *OsBph32* overexpression and BPH infestation are associated with changes in flavonoid-, sugar-, and phenolamide-related metabolic profiles.

### 2.8. Integrated Transcriptome and Metabolome Analysis Suggested Phenylpropanoid Pathway Changes Associated with OsBph32 Overexpression

To explore molecular and metabolic changes associated with *OsBph32*-mediated rice resistance to BPH, integrated transcriptome-metabolome analysis was performed on I-WT vs. I-OE and U-OE vs. I-OE comparisons. Pearson correlation (r > 0.8) and nine-quadrant analysis showed that correlated gene-metabolite pairs were mainly located in quadrants 6 and 9 (Figure S10A,B). The discordance between gene expression and metabolite accumulation suggested that transcript levels and metabolite abundance were not always positively correlated. Correlation heatmaps revealed that strongly correlated metabolites in I-WT vs. I-OE were primarily flavonoids, alkaloids, amino acids and their derivatives, whereas in U-OE vs. I-OE, alkaloids, flavonoids, and organic acids predominated (Figure S10C,D).

**Figure 6:**
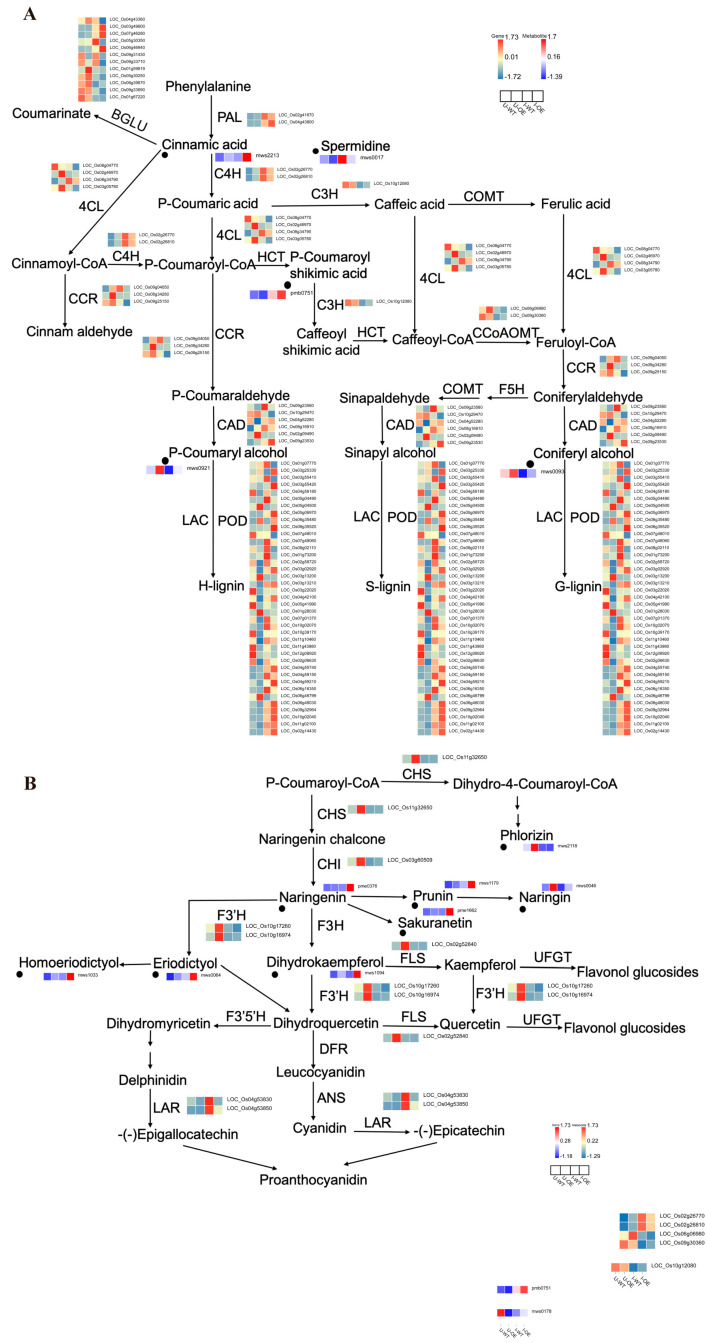
Expression profiles of phenylpropanoid-lignin (**A**) and phenylpropanoid-flavonoid (**B**) biosynthesis genes and metabolites.

Under BPH infestation, both transcripts and metabolites in the phenylpropanoid biosynthesis pathway showed significant differences between OE-*Bph32* and WT plants. Transcriptomic analysis revealed that genes (*PAL*, *C4H*, and *4CL*) encoding the initial enzymes of the phenylpropanoid pathway were significantly upregulated in OE-*Bph32* plants (Figure 6A). Under normal growth conditions, the expression of *4CLL3*, *4CL2*, *4CL5*, and *4CLL1* was enhanced; upon BPH infestation, *PAL3*, *PAL6*, and *C4H* were induced. In the downstream lignin biosynthesis branch, genes encoding *CCR*, *CCoAOMT*, *CAD*, and numerous *PODs* underwent transcriptional reprogramming, with *OsCAD8A* and *OsCAD7* being significantly induced after infestation. Concurrently, the expression levels of multiple *BGLU* family genes (e.g., *BGLU2*, *BGLU19*, *BGLU28*) were significantly elevated upon OE-*Bph32* or infestation. In the flavonoid branch, only a few genes, including *OsCHS1*, *CHI*, *F3’H*, and *FLS*, were differentially expressed and all were upregulated in the OE-*Bph32* plants (Figure 6B).

Metabolomic analysis showed that cinnamic acid, an initial metabolite of the phenylpropanoid pathway, accumulated to high levels in OE-*Bph32* plants in response to BPH feeding. Few differential metabolites were identified in the lignin branch; among them, p-coumaroyl shikimate was markedly enriched in OE-*Bph32* plants after infestation. Although p-coumaryl alcohol and coniferyl alcohol accumulated upon OE-*Bph32* plants, their levels decreased under BPH infestation (Figure 6A). In contrast, the flavonoid branch exhibited a substantial increase in differential metabolites, with naringenin, prunin, sakuranetin, dihydrokaempferol, and eriodictyol showing significantly elevated levels in infested OE-*Bph32* plants (Figure 6B).

In summary, *OsBph32* overexpression enhanced rice resistance to BPH infestation and was associated with coordinated transcriptional and metabolic changes, particularly involving the phenylpropanoid pathway, lignin deposition, and flavonoid biosynthesis.

## 3. Discussion

The present results suggest that *OsBph32*-associated resistance should be considered in the context of local defense at the BPH feeding site rather than as a general stress response alone. BPH is a phloem-feeding insect, and its successful colonization depends on sustained access to sieve tube sap. Previous studies have shown that callose deposition on sieve plates, β-1,3-glucanase-mediated callose degradation, and cell wall reinforcement can strongly influence rice resistance to BPH [12,27]. In OE-*Bph32* plants, BPH resistance was accompanied by enhanced callose deposition, reduced β-1,3-glucanase activity, increased cell wall thickness in phloem-associated tissues, and higher lignin accumulation after infestation. These changes suggest that *OsBph32*-associated resistance may involve structural remodeling of leaf sheath and vascular tissues, thereby limiting sustained BPH feeding. However, not all cell wall-related changes followed the same direction, as pectin content decreased after BPH feeding. This indicates that the cell wall response in OE-*Bph32* plants is better interpreted as dynamic remodeling rather than uniform reinforcement of all wall components.

The phenylpropanoid pathway provides a biochemical link between structural reinforcement and chemical defense because it generates lignin-related metabolites that contribute to cell wall strengthening, as well as flavonoids and other specialized metabolites involved in plant defense responses [31,32]. In this study, OE-*Bph32* plants showed changes in both lignin-related and flavonoid-related branches after BPH infestation, including increased lignin accumulation and higher levels of several flavonoid-related metabolites. Similar enrichment of phenylpropanoid and flavonoid metabolism has also been reported in other rice–BPH resistance systems. For example, integrated transcriptomic and metabolomic analysis of *Bph30*-mediated resistance revealed the involvement of shikimate pathway, phenylpropanoid metabolism, flavonoids, lignin, and IAA-related pathways in BPH resistance [24]. In *BPH14*/*BPH15* pyramiding rice, flavonoid and phenylpropanoid biosynthesis pathways were more strongly altered in the resistant pyramiding line than in the recurrent parent during BPH infestation [33]. In addition, the *OsmiR396*–*OsGRF8*–*OsF3H* module has been shown to mediate rice resistance to BPH through the flavonoid pathway [34]. Together, these studies suggest that phenylpropanoid- and flavonoid-derived metabolism represents a recurrent metabolic feature of rice defense against BPH.

Moreover, studies in other insect-resistant plant systems also support this view. For example, in the wild tomato *Solanum habrochaites*, higher accumulation of phenylpropanoids and flavonoids was associated with insect resistance, and silencing of *Sl4CLL6* reduced the expression of downstream phenylpropanoid-pathway genes and weakened mite resistance [35]. Therefore, the phenylpropanoid-derived metabolic changes observed in OE-*Bph32* plants may contribute to both cell wall-associated structural defense and chemical defense during BPH infestation. Nevertheless, because the present study mainly provides transcriptomic and metabolomic association evidence, further functional analysis of candidate genes in the lignin and flavonoid branches will be needed to determine whether these metabolic changes are required for *OsBph32*-mediated resistance.

The transcriptome data also revealed extensive suppression of photosynthesis-related genes after BPH infestation, with 73 of 76 DEGs in the photosynthetic pathway being downregulated. This pattern is consistent with the concept of a growth–defense trade-off, in which plants reduce investment in growth-associated processes while activating defense under biotic stress [36]. Insect herbivory can also reduce photosynthetic performance through direct tissue damage or indirect physiological changes in remaining tissues. However, the physiological data in this study argue against a simple interpretation of photosynthetic impairment in OE-*Bph32* plants. After BPH feeding, OE-*Bph32* plants showed smaller decreases in chlorophyll b and total chlorophyll, maintained relatively higher PFK activity, and accumulated more starch in leaf sheaths than WT plants. Thus, the downregulation of photosynthesis-related genes may represent defense-oriented adjustment of carbon metabolism rather than passive damage to the photosynthetic system.

This interpretation helps reconcile the apparent inconsistency between transcriptomic and physiological data. On the one hand, suppression of photosynthesis-related transcripts may reduce growth-associated carbon expenditure during defense activation. On the other hand, higher starch accumulation and altered sugar metabolism suggest that carbon may be temporarily stored or repartitioned in OE-*Bph32* plants during BPH attack. Because starch and soluble sugars can provide carbon skeletons and energy for secondary metabolism, cell wall biosynthesis, and defense responses, this carbon metabolic adjustment may support the formation of both structural and chemical defense outputs. Nevertheless, this remains a working interpretation. Measurements of photosynthetic rate, chlorophyll fluorescence and source–sink transport will be required to determine whether *OsBph32* actively redirects carbon flow toward defense-related pathways.

Although these results provide a more detailed view of *OsBph32*-associated defense responses, the current evidence should be interpreted cautiously. First, this study mainly used overexpression lines, and loss-of-function mutants, complementation lines, or near-isogenic materials will be needed to confirm the native function of *OsBph32*. Second, transcriptome–metabolome integration identifies coordinated changes between gene expression and metabolite accumulation, but does not establish direct regulatory relationships. Third, although the SCR domain of *OsBph32* suggests a potentially distinct type of resistance-associated protein, its biochemical function and interacting partners remain unknown. Therefore, future studies should focus on identifying *OsBph32*-interacting proteins, determining whether *OsBph32* acts at the plasma membrane, apoplast, or cell wall interface, and testing candidate genes in the lignin, flavonoid, and carbon metabolism branches. Such experiments will be necessary to determine whether the observed structural and metabolic changes are direct consequences of *OsBph32* activity or downstream responses associated with enhanced resistance.

## 4. Materials and Methods

### 4.1. Plants and Insects

Indica rice material Ptb33 was used in this experiment to clone cDNA fragments to construct the overexpression vector and 9311 was used as the recipient variety to construct OE-*Bph32* plants. As the insect source, the BPH biotype 1 insects were reared on the susceptible rice variety Taichung Native 1 (TN1) under controlled environmental conditions (26 ± 0.5 °C, 16 h-light/8 h-dark cycle).

### 4.2. Vector Construction

The cloning and construction were performed using Gateway technology (Invitrogen, Carlsbad, CA, USA). For the construction of the pGWB5-*Bph32* construct, full-length *Bph32* cDNA was amplified by RT-PCR from Ptb33 total RNA. First, the cDNA was inserted into the pDONR201 vector (Thermo Fisher Scientific, Waltham, MA, USA) by the BP reaction (Gateway^®^ BP Clonase^TM^ Enzyme Mixtures, Thermo Fisher Scientific, Waltham, MA, USA). After verification by sequencing, they were transferred into the final vector pGWB5 through the Gateway LR recombinase reaction (Gateway^®^ LR Clonase^TM^ Enzyme Mixtures, Invitrogen, Thermo Fisher Scientific, Waltham, MA, USA). The resulting plasmid was used for the transformation of *Agrobacterium tumefaciens* strain EHA105. All the primer sequences for the constructs are indicated in Supplementary Table S1.

### 4.3. Rice Genetic Transformation

The constructed vector was sent to the Wuhan Boyuan Biotechnology Co., Ltd. (Wuhan, China) for genetic transformation. Then, 24 transgenic plants were obtained. A hygromycin resistance gene was used to detect whether the target genes were transferred into the rice material. In addition, verification primers were designed by comparing the sequence of the gene inserted into the vector and the target fragment was amplified by PCR to detect whether the target gene was integrated into the vector and correctly transferred into the rice. The primers Hyg and 35S-*Bph32* are shown in Supplementary Table S1. Positive transgenic plants were identified with PCR and sequence analysis. Three independent lines of transgenic T2 generation materials were selected, and the protein expression and mRNA relative expression levels were detected by the Western blot and qRT-PCR, respectively. Among the three independent lines, OE-*Bph32*-3 showed the highest expression level and was therefore selected as the representative high-expression line for subsequent physiological, biochemical, transcriptomic, and metabolomic analyses, unless otherwise stated.

### 4.4. Western Blot Analysis

Rice leaf sheaths were ground into fine powder in liquid nitrogen, and total proteins were extracted using a plant protein extraction kit (Yeasen Biotechnology, Shanghai, China, #20131ES06) according to the manufacturer’s instructions. The homogenate was centrifuged at 12,000× *g* for 10 min at 4 °C, and the supernatant was collected as the total protein extract. Protein samples were denatured at 98 °C for 10 min. Equal amounts of protein (30 µg per lane) were separated by SDS-PAGE and subsequently transferred onto PVDF membranes (Millipore, Burlington, MA, USA). The membranes were blocked with 5% (*w*/*v*) non-fat dry milk in TBST buffer (Tris-buffered saline containing 0.1% Tween-20) for 1 h at room temperature, and then incubated overnight at 4 °C with primary antibodies against GFP (1:2000, ABclonal Technology, Wuhan, China) and Actin (1:5000, ABclonal Technology, Wuhan, China). After three washes with TBST, the membranes were incubated with HRP-conjugated secondary antibody (1:10,000) for 1 h at room temperature. Protein signals were detected using an ECL chemiluminescent substrate and visualized with a ChemiDoc imaging system (Bio-Rad Laboratories, Hercules, CA, USA).

### 4.5. Identification of Resistance to BPH by Damage Degree of Rice Seedlings

The transgenic homozygous lines of T2 generation of each independent T0 generation plant were used as the material to test the resistance to BPH. When the rice grew to the two leaves and one heart stage, the rice seedlings were infested with 2–3 instar BPH nymphs at 8 nymphs per plant. When the dead seedling rate of the control wild-type 9311 reached approximately 95%, the rice plants were photographed and scored. Each batch of material contained 60 plants, with 20 plants constituting one biological replicate, for a total of three replicates. If two of the three biological replicates were medium resistant, the material was identified as medium resistant. The evaluation followed the standards of the International Rice Research Institute (IRRI).

The individual plant grading evaluation criteria for identification of BPH resistance at seedling stage of rice are listed below:


**Resistance Level**

**Seedling Damage Degree**
0The plant was unharmed3One to two leaves were yellowing, and the yellowing parts did not exceed 50% of the leaf area; or the first leaf was withered, and the yellowing part of the second leaf did not exceed 30% of the leaf area5Two or three leaves were markedly yellowed, and the yellowing part exceeded 50% of the leaf area; or one or two leaves were withered7Three or four leaves were withered, but the plant was not yet dead9The plant died

Grading standards for identification and grading of rice seedling resistance to BPH:


**Mean Value of Resistance Grade**

**Rice Resistance Level**
0 < average ≤ 2.0 High resistance2 < average ≤ 4.0Resistance 4 < average ≤ 5.5Medium resistance5.5 < average ≤ 8.0Sensitive 8< averageHigh sensitive

### 4.6. Identification of Resistance to BPH by BPH Weight Gain Method

The positive homozygous transgenic material of the T2 generation to be identified was sown in 3 cups per group with 5 seeds per cup. Female BPH with body weight between 1.8 and 2.7 mg were selected as identification insects. When the seedlings grew to the 6–7 leaf stage, only two of the seedlings with consistent growth were retained for identification; the identification was repeated 3 times. A BPH that had been weighed and recorded as A were put into the wax bag, and the wax bag was tied to the plant base above 1 cm from the ground. Each rice plant could be tied by two wax bags. After the BPH in the wax bag fed on rice for 48 h, its weight was measured and recorded as B. The weight gain rate of the BPH was Weight gain rate (%) = [(B − A)/A] × 100. The BPH resistance level of rice was evaluated according to the weight changes in female BPH after feeding on rice plants for 48 h. The rating criteria were as follows:


**Average Weight Gain Rate**

**Rice Resistance Level**
0 < average ≤ 20 High resistance20< average ≤ 40Resistance 40< average ≤ 60Medium resistance60 < average ≤ 80Sensitive average >80High sensitive

### 4.7. RNA Extraction and RNA-Seq Sequencing

The rice leaf sheaths of the plants including U-WT, U-OE, I-WT and I-OE were collected and frozen in liquid nitrogen, and stored at −80 °C until use. Total RNA was isolated from rice leaf sheaths by TRIzol Reagent (Thermo Fisher Scientific, Waltham, MA, USA) according to the manufacturer’s instructions. Nanodrop 2000C Spectrophotometer (Thermo Fisher Scientific, Waltham, MA, USA) was used to detect RNA concentration and purity, to ensure sample concentration ≥50 ng/μL and sample purity: OD _260/280_ ≈ 2.0, OD _260/230_ between 2.0 and 2.2, with a peak at 260 nm. Subsequently, the leaf samples were sent to Biomarker Technologies Corporation (Beijing, China) for transcriptome sequencing. For each treatment, leaf sheaths from 30 individual plants were pooled as one composite sample for RNA-seq analysis. The raw sequencing data generated in this study have been deposited in the NCBI Sequence Read Archive under BioProject accession number PRJNA1489418.

### 4.8. Quantitative Real-Time RT-PCR (qRT-PCR) Assay

Total RNA was isolated using the TRIzol method from leaf sheaths collected at the four-leaf-and-one-heart stage for the I-WT vs. I-OE and U-OE vs. I-OE comparisons. After verifying RNA purity and integrity, the HIScriptIII 1st Strand cDNA Synthesis Kit (+gDNA wiper; Vazyme, Nanjing, China) was used to reverse-transcribe the RNA into complementary DNA (cDNA). Primer sequences were designed using Premier 5.0 and synthesized by Tsingke Biotechnology (Beijing, China). The primer sequences used are shown in Supplementary Table S1. qRT-PCR was performed using Hieff qPCR SYBR Green Master Mix (Yeasen Biotechnology, Shanghai, China) according to the manufacturer’s instructions. Using *OsActin* (LOC_Os03g50885) as an internal reference, the relative gene expression levels were determined by the 2^−∆∆Ct^ method. Three biological replicates were performed, with each replicate comprising leaf sheaths from 10 plants. Three technical replicates were performed for each biological replicate.

### 4.9. Histochemical Detection of ROS

Rice seeds were soaked overnight and germinated at 37 °C for 24 h. The pre-germinated seeds were evenly spread in a rectangular box lined with three layers of wet gauze, covered with black cloth, and cultured in the dark at 28 °C for 10–14 days. Rice protoplasts were isolated from etiolated basal stem tissues and used for transient expression assays. The OsBph32-GFP construct was introduced into rice protoplasts via PEG4000-mediated transformation and incubated for 16–22 h. After transformation, cells were centrifuged at 350×g, the supernatant was discarded, and the pellets were resuspended in W1 solution and allowed to recover in W5 solution for 5 h. Cell density was adjusted to 2 × 10^6^ cells/mL with W1 solution. The cells were then exposed to strong light (100–150 µmol·s^−1^·m^−2^) for 1 h, followed by incubation with an equal volume of H_2_DCFDA loading solution (10 μM) in the dark for 30 min. After two washes with W5 solution to remove residual dye, fluorescence was observed and imaged using a TCS SP8 confocal laser scanning microscope (Leica Microsystems, Wetzlar, Germany; excitation 450–490 nm). Fluorescence intensity was quantified using ImageJ software (version 1.54g) from 15 randomly selected independent protoplasts for each treatment.

### 4.10. Determination of H_2_O_2_ Content and Antioxidant Enzyme Activities

In this experiment, WT and OE-*Bph32* were used as the samples to be tested, 5–10 rice leaf sheaths from each treatment were mixed as one biological replicate for detection after BPH infection at 0, 12, 24 and 48 h. Each biological replicate was subjected to three technical replicates. Three biological replicates were carried out. The Superoxide Dismutase (SOD) Activity Detection Kit (AKAO001C, Boxbio, Beijing, China), the Catalase (CAT) Activity Assay Kit (AKAO003-1U, Boxbio, Beijing, China) and the Peroxidase (POD) Activity Detection Kit (AKAO004C, Boxbio, Beijing, China) were used to detect the activity of SOD, CAT and POD, respectively. H_2_O_2_ content was determined using a Hydrogen Peroxide (H_2_O_2_) Content Assay Kit (AKAO009M, Boxbio, Beijing, China) according to the manufacturer’s instructions. All procedures were performed according to the manufacturers’ instructions.

### 4.11. Metabolome Analysis

Rice leaf sheaths were collected, immediately frozen in liquid nitrogen, and stored at −80 °C for later use. For sample extraction, the samples were vacuum freeze-dried using a Scientz-100F freeze dryer (Ningbo Scientz Biotechnology Co., Ltd., Ningbo, China). The dried samples were then ground into powder using an MM 400 mixer mill (Retsch GmbH, Haan, Germany) for 1.5 min at 1.5 Hz. Subsequently, 100 mg of powder was weighed and extracted overnight at 4 °C with 1.2 mL of 70% methanol. After centrifugation at 12,000× *g* for 10 min, the supernatant was filtered through a 0.22 µm microporous membrane. The analysis was performed by Metware Biotechnology Co., Ltd. (Wuhan, China). The rice leaf sheaths of U-WT, U-OE, I-WT and I-OE were selected as the samples, each containing 10 mixed rice leaf sheaths, a total of three biological replicates, each sample had three technical replicates.

### 4.12. Lignin Determination

The phenolic hydroxyl group in lignin has a characteristic absorption peak at 280 nm after acetylation, and the absorbance value at 280 nm is positively correlated with the lignin content. Lignin content was determined by the acetyl bromide method according to the manufacturer’s instructions (Solarbio, Beijing, China).

The wild-type (WT) plants and OE-*Bph32* plants were sown at 5–8 plants per cup and only 2–3 plants in good growth condition were kept before releasing the insects. Eight to ten BPH were placed on each plant as the treatment. The BPH feeding site was restricted to within 5 cm above the base of the rice plant. Rice leaf sheaths from WT and OE-*Bph32* plants under uninfested conditions and after 24 h of BPH infestation were sampled. Afterwards, the leaf sheaths were cut into 0.5 cm long segments with a single-sided blade and fixed immediately with pre-cooled 70% FAA fixation solution (38% formaldehyde: acetic acid: 70% ethanol = 5:5:90 [*v*/*v*/*v*]). The samples were completely immersed in the fixation solution, vacuumed and fixed for 15 min. Then, they were slowly deflated and vacuumed twice until the samples sank completely. Next, the fixative was replaced and samples were incubated in fixative for 1–2 days at 4 °C. Then, dehydration was carried out with different concentrations of ethanol. Subsequently, the leaf sheaths were dehydrated and embedded in paraffin, and then cut into 5–10 μm thick wax strips with a microtome (Thermo Fisher Scientific, Waltham, MA, USA). Different gradient concentrations of xylene and ethanol were used for dewaxing and rehydration. The rehydrated material was stained in the phloroglucinol-hydrochloric acid solution for 2 min and placed under the white light channel of an Olympus BX51 (Olympus Corporation, Tokyo, Japan) microscope to observe lignin.

### 4.13. Detection of Cell Wall Components and Carbohydrate Content

Rice leaf sheaths of WT and OE-*Bph32* plants under uninfested conditions and after 24 h of BPH infestation were collected for the detection of cell wall components and carbohydrate contents. The contents of cellulose, hemicellulose, and pectin were determined using assay kits from Beijing Boxbio Science & Technology Co., Ltd. (Boxbio, Beijing, China) according to the manufacturer’s instructions. Glucose and fructose contents were determined using assay kits from Nanjing Jiancheng Bioengineering Institute (Nanjing, China) according to the manufacturer’s instructions. The decrease in glucose or fructose content was calculated as the value under uninfested conditions minus the value after BPH infestation.

### 4.14. Observation of Callose Deposition

Rice leaf sheaths were fixed in a solution of ethanol and acetic acid (3:1, *v*/*v*) for 5 h, with frequent changes in the fixative to ensure complete tissue fixation. The samples were then rehydrated sequentially in 70% ethanol for 2 h, 50% ethanol for 2 h, and left in distilled water overnight. After rinsing three times with distilled water, the leaf sheaths were treated with 10% sodium hydroxide for 1 h to clarify the tissues. Following four rinses with distilled water, the specimens were incubated for 4 h in 150 mM K_2_HPO_4_ (pH 9.5) containing 0.01% aniline blue (Sigma-Aldrich, St. Louis, MO, USA). The stained leaf sheaths were mounted on glass slides, and callose deposits were immediately observed under UV light using an Olympus BX51 (Olympus Corporation, Tokyo, Japan) fluorescence microscope.

### 4.15. β-1,3-glucanase (β-1,3-GA) Activity Assay

β-1,3-glucanase (β-1,3-GA) hydrolyzes laminarin by cleaving internal β-1,3-glucosidic bonds, thereby generating reducing ends. The enzyme activity is determined by calculating the rate of reducing sugar production. Rice leaf sheath samples were collected from WT and OE-*Bph32* plants at 0, 3, 6, 12, 24, 36, 48, 60, and 72 h after BPH infestation for β-1,3-GA activity measurement. In this experiment, the β-1,3-GA activity was measured using an assay kit (Solarbio, Beijing, China). The assay was performed according to the manufacturer’s instructions.

### 4.16. Determination of Enzyme Activities and Metabolite Contents

The activities of α-amylase (α-AL) and phosphofructokinase (PFK) in rice leaf sheaths were measured at 0, 3, 6, 12, 24, 36, 48, 60, and 72 h after BPH infestation using commercial assay kits (Solarbio, Beijing, China). The determinations were performed strictly following the manufacturer’s instructions (Products No. BC0615 for α-AL and BC0530 for PFK).

Glucose, fructose, chlorophyll a, chlorophyll b, and total chlorophyll contents were measured using commercial assay kits from Nanjing Jiancheng Bioengineering Institute (Nanjing, China) and Beijing Boxbio Science & Technology Co., Ltd. (Beijing, China), according to the manufacturers’ instructions. For glucose, fructose, and chlorophyll measurements, rice leaf sheaths were collected from WT and OE-*Bph32* plants under uninfested conditions and after 24 h of BPH infestation. The decrease in glucose, fructose, or chlorophyll content was calculated as the value under uninfested conditions minus the value after BPH infestation.

### 4.17. Starch Content Assay and KI-I_2_ Staining for Starch Granules

For starch content measurement and KI-I_2_ staining, rice leaf sheaths were collected from WT and OE-*Bph32* plants under uninfested conditions and after 24 h of BPH infestation. Starch content was measured using a commercial assay kit (Solarbio, Beijing, China). The principle involves removing soluble sugars with 80% ethanol, followed by acid hydrolysis of starch to glucose. The released glucose reacts with anthrone reagent to form a colored compound, the absorbance of which is measured at a specific wavelength using a spectrophotometer. The detailed procedure was strictly followed according to the manufacturer’s instructions (Product No. BC0700).

The staining procedure was as follows: Rice leaf sheaths were collected and fixed in 70% FAA fixative (formalin: glacial acetic acid: 70% ethanol = 5:5:90, *v*/*v*/*v*). After dehydration through a graded ethanol series and paraffin embedding, the samples were sectioned transversely to a thickness of 5–10 µm using a microtome. The sections were mounted on glass slides, dewaxed, and rehydrated. To visualize starch granules in the leaf sheath cross-sections, the rehydrated sections were immersed in a 3% (*w*/*v*) KI-1% (*w*/*v*) I_2_ staining solution for 1 min, briefly rinsed with distilled water, and then observed and imaged under a light microscope. Starch granules appeared purplish-blue.

### 4.18. Transmission Electron Microscopy Observation

Rice leaf sheaths were excised and cut into small pieces of approximately 1–2 mm. The samples were immediately immersed in freshly prepared prefixation solution containing 2% glutaraldehyde in 100 mM phosphate buffer (pH 7.4). Vacuum infiltration was applied until the tissues completely sank into the solution. The samples were then transferred to fresh fixative and kept at 4 °C for 4–6 h. After fixation, the tissues were rinsed five times with 100 mM phosphate buffer (pH 7.4), 20 min each time. Dehydration was carried out through a graded ethanol series: 15%, 30%, 50%, and 70% ethanol for 30 min each, followed by 80%, 85%, 90%, and 95% ethanol for 20 min each. The samples were then treated with absolute ethanol for 45 min (repeated twice).

Subsequently, the samples were incubated in a mixture of absolute ethanol and propylene oxide (1:1) for 30 min, followed by two changes of pure propylene oxide for 30 min each. Gradual infiltration with Spurr resin was performed using propylene oxide: Spurr resin mixtures at ratios of 3:1 (4 h), 2:1 (4 h), 1:1 (12 h), 1:2 (12 h), and 1:3 (12 h). Finally, the samples were infiltrated with two changes of pure Spurr resin (12 h each, or overnight).

The tissues were placed into embedding molds filled with fresh Spurr resin and polymerized in an oven at 40 °C for 8 h, then at 60 °C for 1–2 days. Ultrathin sections (80 nm thick) were cut using an ultramicrotome (Leica Microsystems, Wetzlar, Germany), mounted on copper grids, and post-stained with uranyl acetate and lead citrate. The samples were observed under a transmission electron microscope (JEM-1010, JEOL Ltd., Tokyo, Japan).

### 4.19. Rice Grain Quality Analysis

For rice grain quality assessment, the chalkiness rate of milled rice was analyzed using a Wanshen SC-E rice appearance quality scanner (Hangzhou Wanshen Detection Technology Co., Ltd., Hangzhou, China), with three replicates per variety. The amylose content of milled rice was determined using a DA 7250 near-infrared analyzer (Perten Instruments AB, Hägersten, Sweden), with three replicates per variety. For observation of starch granule morphology, mature milled rice grains were attached to conductive adhesive, sputter-coated with gold, and mounted on specimen holders. The samples were then placed into the chamber of a scanning electron microscope (XL30 ESEM, Philips, Eindhoven, The Netherlands), vacuumed, and observed under appropriate accelerating voltage to examine their cross-sectional morphology.

### 4.20. Statistical Analysis

Data are presented as means ± SD unless otherwise indicated. Statistical analyses were conducted using GraphPad Prism 10.4.2 (GraphPad Software, San Diego, CA, USA). Student’s *t*-test was used for comparisons between two groups. One-way ANOVA followed by Tukey’s multiple comparisons test was used for comparisons among more than two groups. For experiments involving genotype and BPH treatment status, data were analyzed by two-way ANOVA followed by Tukey’s multiple comparisons test. For time-course experiments, data were analyzed by two-way ANOVA with genotype and treatment time as factors, followed by Sidak’s multiple comparisons test for comparisons between OE-*Bph32* and WT plants at each time point. Differences were considered statistically significant at *p* < 0.05. Different letters indicate significant differences among groups, and asterisks indicate significant differences between the indicated groups: *, *p* < 0.05; **, *p* < 0.01; and ***, *p* < 0.001.

## Data Availability

The RNA-seq raw data generated in this study have been deposited in the NCBI Sequence Read Archive under BioProject accession number PRJNA1489418. All data supporting the findings of this work are included within the article and its Supplementary Materials. Additional information is available from the corresponding author upon reasonable request.

## Supplementary Materials

The following supporting information can be downloaded at: https://www.mdpi.com/article/10.3390/plants15142132/s1, Figure S1: Molecular identification of OE-*Bph32* plants and function analysis of BPH resistance. A: The schematic diagram of *OsBph32* overexpression vector. B: Western blot analysis of OsBph32-GFP protein expression in OE-Bph32 and WT plants using an anti-GFP antibody. Actin was used as the loading control. C: qRT-PCR detection of transgenic plants OE-*Bph32*. D: The changes in the contents of H_2_O_2_. E: The changes in the activities of SOD, CAT and POD in rice leaves at 0, 12, 24 and 48 h after BPH feeding, respectively. The asterisk indicated the significant difference between OE-*OsBph32* and WT plants. F: GNS family genes expression level before and after BPH infestation in WT and OE-*Bph32* plants detected by qRT-PCR. G: The changes of β-1,3-GA enzyme activity between OE-*Bph32* and WT plants after BPH feeding. The time points were 0, 3, 6, 12, 24, 36, 48, 60, and 72 h after BPH infestation. Statistical significance in C–F was analyzed by Student’s t-test. Statistical significance in G was analyzed by two-way ANOVA followed by Sidak’s multiple comparisons test. The asterisk indicated the significant difference between OE-*Bph32* plants and WT plants (*, *p* < 0.05; **, *p* < 0.01; ***, *p* < 0.001); Figure S2: Different expression genes (DEGs) before and after BPH infestation. A: Venn map of DEGs. B: Volcano map of DEGs between BPH-infested WT and OE-*Bph32* plants (I-WT vs. I-OE). C: Volcano plot of DEGs in OE-*Bph32* plants before and after BPH infestation (U-OE vs. I-OE); Figure S3: MapMan overview of DEGs in OE-*Bph32* plants before and after BPH infestation. A: DEGs related to protein modification, protein degradation, and plant hormone pathways. B: DEGs related to primary metabolism. C: DEGs related to secondary metabolism. D: Overview of defense-related pathways in OE-*Bph32* plants after BPH infestation compared with uninfested plants. Each square represents a DEG. Red indicates induced gene expression, and blue indicates repressed gene expression; Figure S4: GO annotation and KEGG pathway analysis of DEGs between OE-*Bph32* and WT plants after BPH infestation. A: GO terms annotation. B: KEGG pathway enrichment analysis. C: Heat map of DEGs in selected metabolic pathways; Figure S5: MapMan overview of DEGs between BPH-infested OE-*Bph32* and WT plants. A: DEGs related to protein modification, protein degradation, and plant hormone pathways. B: DEGs related to primary metabolism. C: DEGs related to secondary metabolism. D: Overview of defense-related pathways in OE-*Bph32* and WT plants after BPH infestation. Each square represents a DEG. Red indicates induced gene expression, and blue indicates repressed gene expression; Figure S6: Validation of RNA-seq results by qRT-PCR. A: Linear regression analysis between qRT-PCR and RNA-seq log_2_ fold-change values for 10 selected DEGs in I-WT vs. I-OE. B: Linear regression analysis between qRT-PCR and RNA-seq log_2_ fold-change values for 10 selected DEGs in U-OE vs. I-OE. Each point represents one validated DEG. The R^2^ values indicate the consistency between qRT-PCR and RNA-seq data. Standard locus IDs and primers of the validated genes are listed in Table S1; Figure S7: Effect of *OsBph32* overexpression on grain quality. A: Appearance quality of milled rice. B: Scanning electron microscopy observation of cross section of milled rice. Scale bars = 20 µm. C: Chalkiness percentage of milled rice. D: Amylose content of milled rice. Statistical significance was analyzed by Student’s t-test. Asterisks indicate significant differences between OE-*Bph32* and WT plants: **, *p* < 0.01; Figure S8: Evaluation of metabolome data. A: Number of differential abundance metabolites (DAMs). B: Venn diagram of DAMs. C: Principal component analysis of the four sample groups. D: Correlation evaluation of the four sample groups. E: Cluster analysis of DAMs in the four sample groups; Figure S9: K-means clustering of differentially accumulated metabolites across the four sample groups; Figure S10: Integrated transcriptome–metabolome correlation analysis. A: Nine-quadrant analysis of gene–metabolite correlations in I-WT vs. I-OE. B: Nine-quadrant analysis of gene–metabolite correlations in U-OE vs. I-OE. C: Correlation coefficient clustering heatmap of gene–metabolite pairs in I-WT vs. I-OE. D: Correlation coefficient clustering heatmap of gene–metabolite pairs in U-OE vs. I-OE. In panels A and B, each point represents a gene–metabolite pair. The x-axis indicates the log_2_ fold change of genes, and the y-axis indicates the log_2_ fold change of metabolites. Gene–metabolite pairs in different quadrants represent different correlation patterns between transcript and metabolite changes. Panels C and D show the correlation coefficients between differentially expressed genes and differential abundance metabolites, with metabolite classes indicated by different colors; Table S1: Primers used in this study; Table S2: Statistics of DEGs; Table S3: List of DAMs (Fold change > 5).
