# Supplementary material for: OsBph32 Contributes to Coordinated Cell Wall and Metabolic Responses in Rice Resistance to Brown Planthopper
Source: Plants (Basel). 2026 Jul 10;15(14):2132. doi: 10.3390/plants15142132 (PMC13415291; doi:10.3390/plants15142132)
Supplement: Supplementary file 1 [file plants-15-02132-s001.zip › plants-4413839-supplementary.pdf]

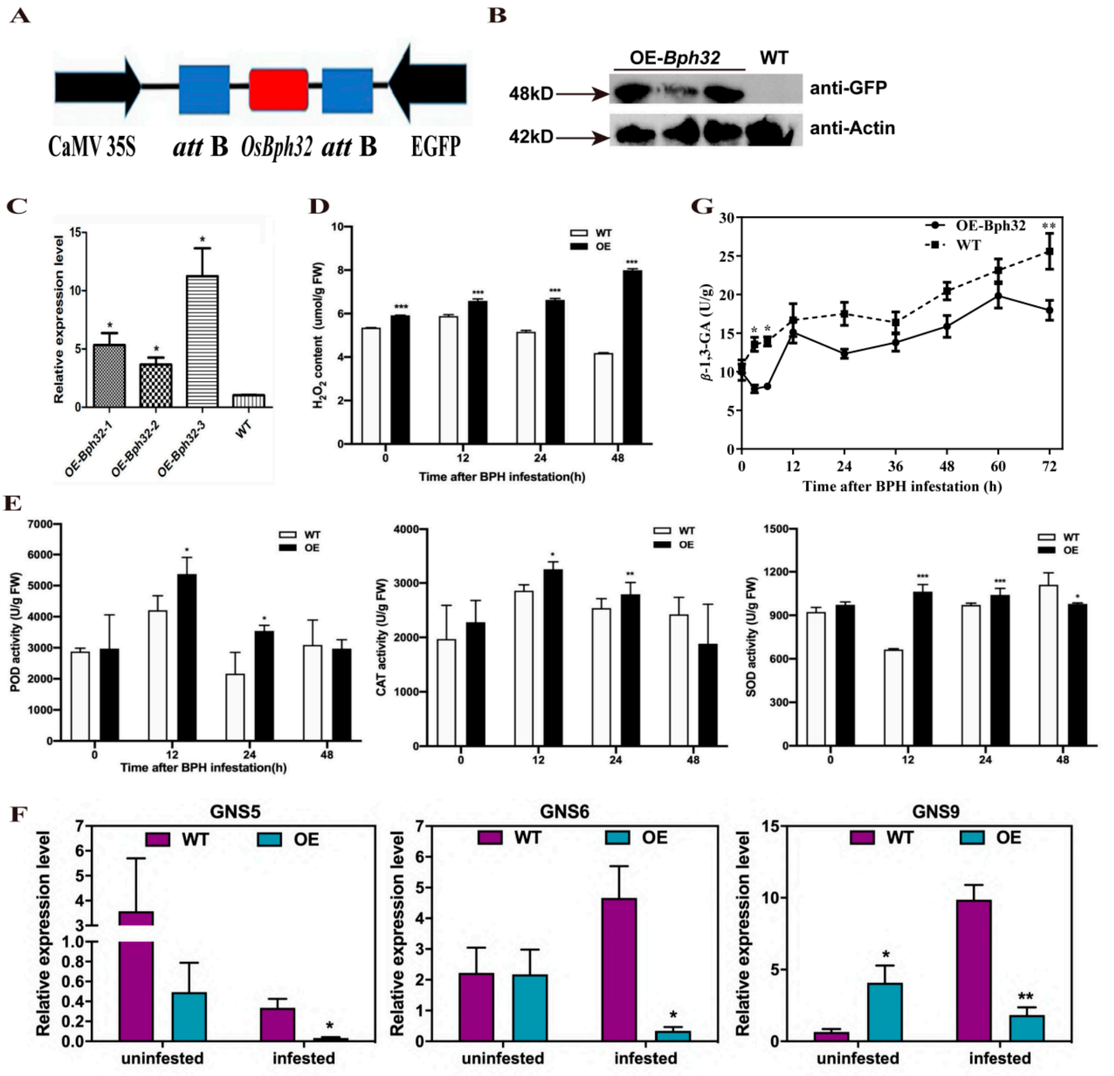

Figure S1. Molecular identification of OE-*Bph32* plants and function analysis of BPH resistance. A: The schematic diagram of *OsBph32* overexpression vector. B: Western blot analysis of OsBph32-GFP protein expression in OE-Bph32 and WT plants using an anti-GFP antibody. Actin was used as the loading control. C: qRT-PCR detection of transgenic plants OE-*Bph32*. D: The changes in the contents of H_2O_2. E: The changes in the activities of SOD, CAT and POD in rice leaves at 0, 12, 24 and 48 h after BPH feeding, respectively. The asterisk indicated the significant difference between OE-*OsBph32* and WT plants. F: GNS family genes expression level before and after BPH infestation in WT and OE-*Bph32* plants detected by qRT-PCR. G: The changes of β-1,3-GA enzyme activity between OE-*Bph32* and WT plants after BPH feeding. The time points were 0, 3, 6, 12, 24, 36, 48, 60, and 72 h after BPH infestation. Statistical significance in C–F was analyzed by Student's t-test. Statistical significance in G was analyzed by two-way ANOVA followed by Sidak's multiple comparisons test. The asterisk indicated the significant difference between OE-*Bph32* plants and WT plants (*, $P < 0.05$; **, $P < 0.01$; ***, $P < 0.001$).

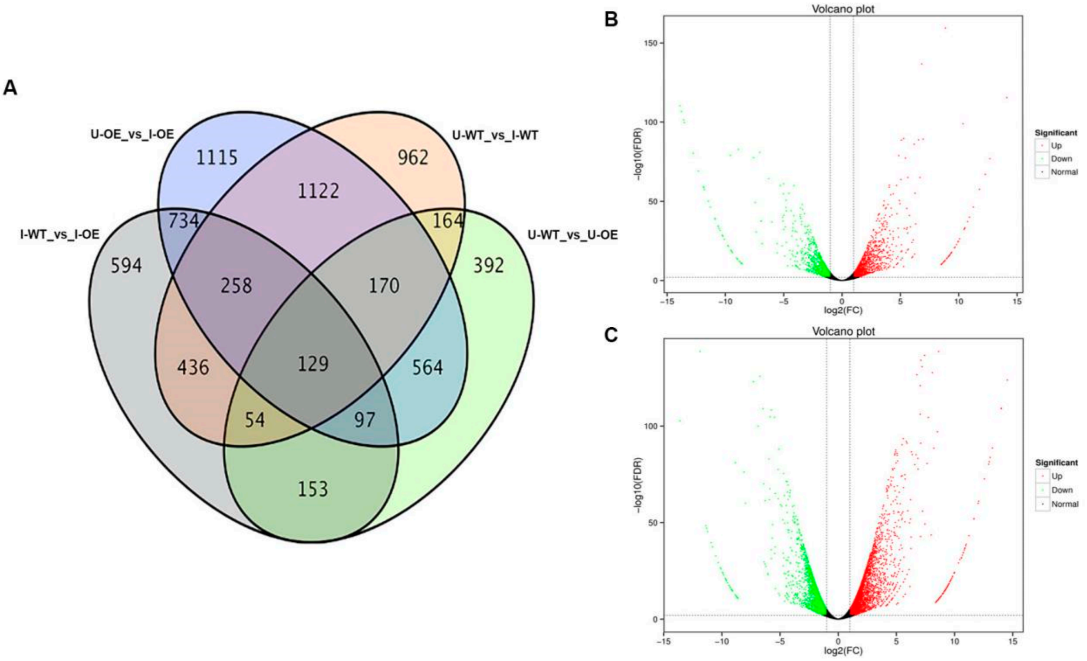

Figure S2 Different expression genes (DEGs) before and after BPH infestation. A: Venn map of DEGs. B: Volcano map of DEGs between BPH-infested WT and OE-*Bph32* plants (I-WT vs. I-OE). C: Volcano plot of DEGs in OE-*Bph32* plants before and after BPH infestation (U-OE vs. I-OE).

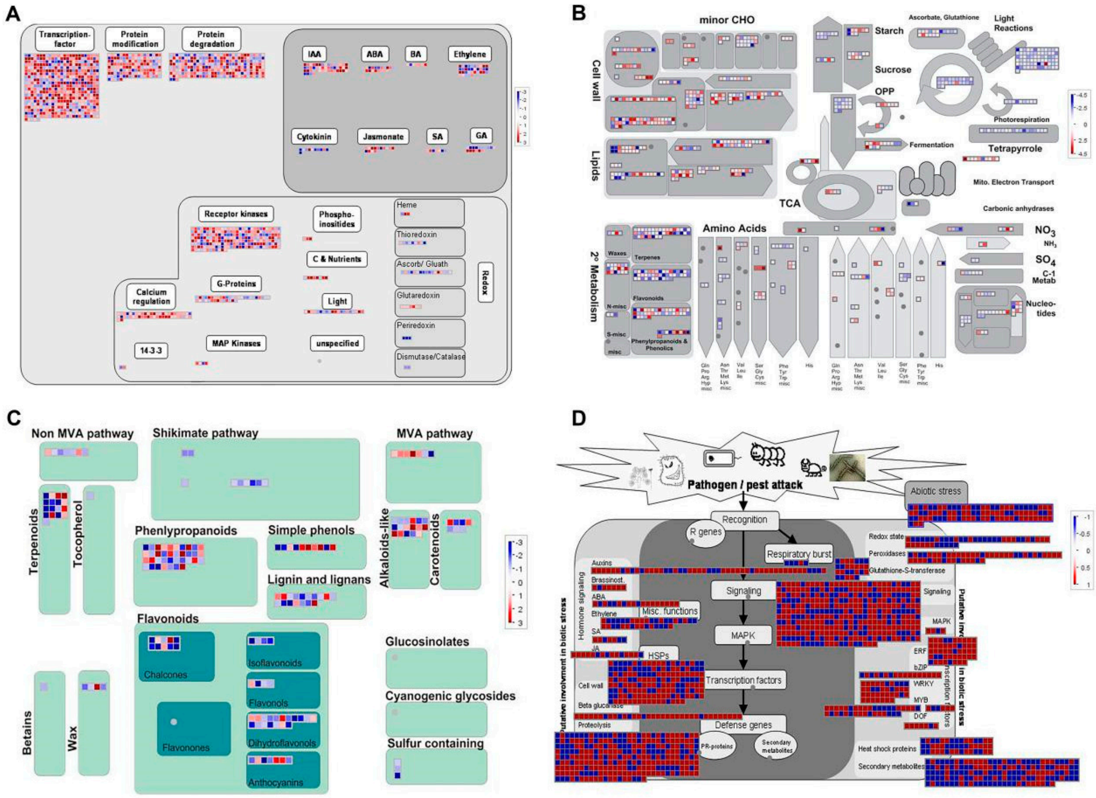

Figure S3. MapMan overview of DEGs in OE-*Bph32* plants before and after BPH infestation. A: DEGs related to protein modification, protein degradation, and plant hormone pathways. B: DEGs related to primary metabolism. C: DEGs related to secondary metabolism. D: Overview of defense-related pathways in OE-*Bph32* plants after BPH infestation compared with uninfested plants. Each square represents a DEG. Red indicates induced gene expression, and blue indicates repressed gene expression.

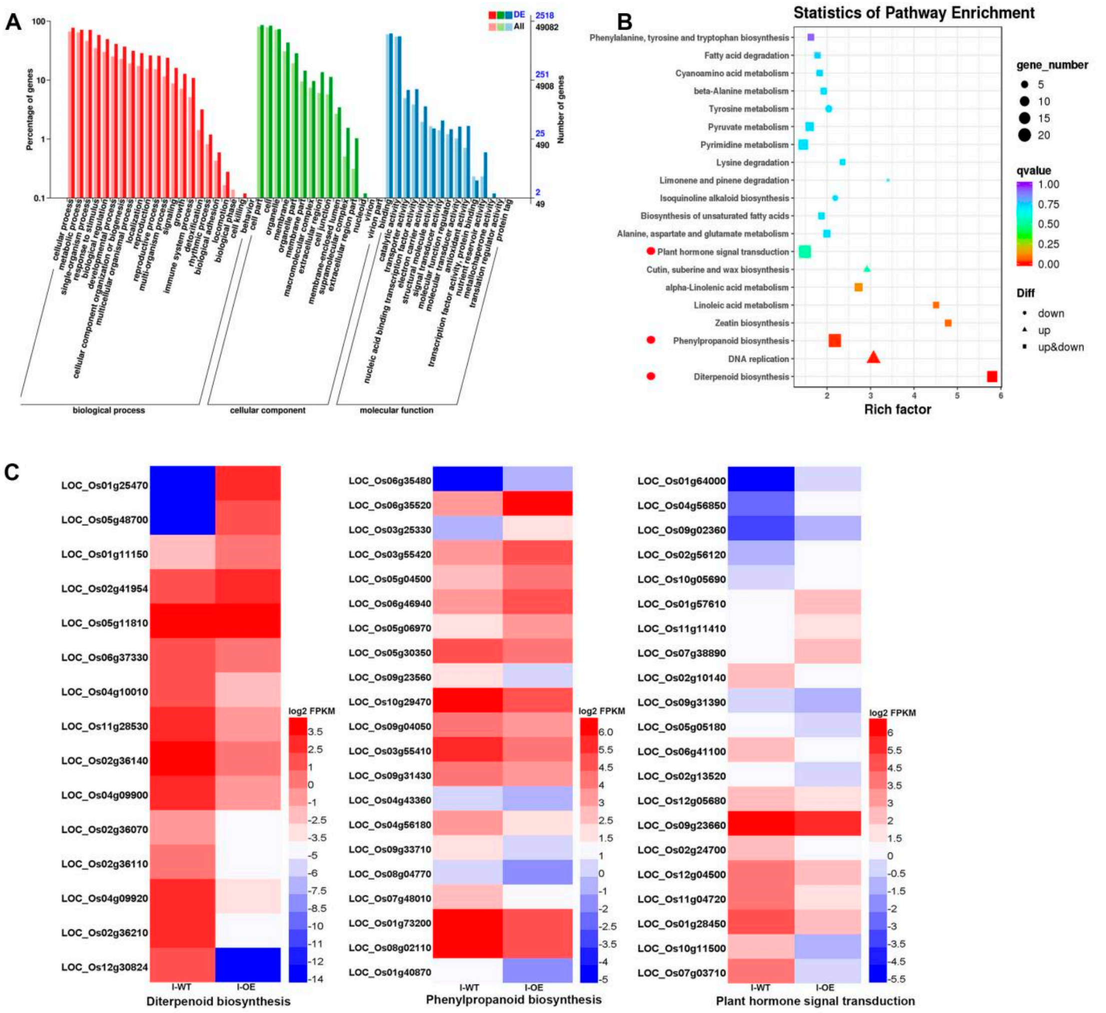

Figure S4. GO annotation and KEGG pathway analysis of DEGs between OE-*Bph32* and WT plants after BPH infestation. A: GO terms annotation. B: KEGG pathway enrichment analysis. C: Heat map of DEGs in selected metabolic pathways.

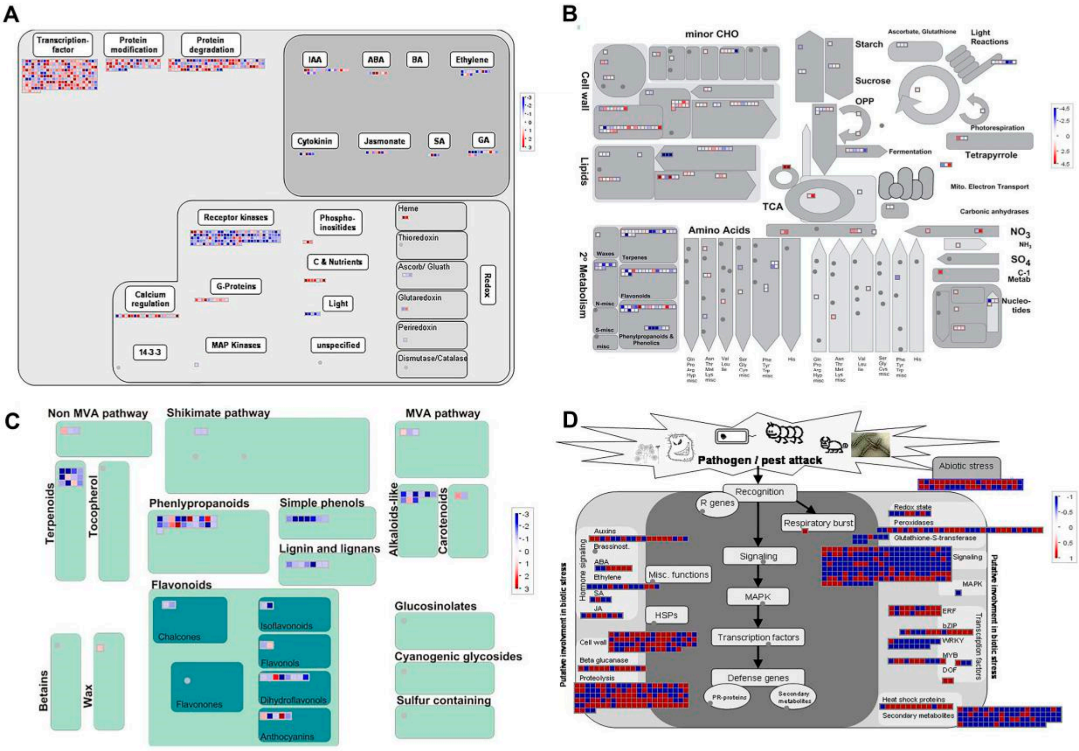

Figure S5. MapMan overview of DEGs between BPH-infested OE-*Bph32* and WT plants. A: DEGs related to protein modification, protein degradation, and plant hormone pathways. B: DEGs related to primary metabolism. C: DEGs related to secondary metabolism. D: Overview of defense-related pathways in OE-*Bph32* and WT plants after BPH infestation. Each square represents a DEG. Red indicates induced gene expression, and blue indicates repressed gene expression.

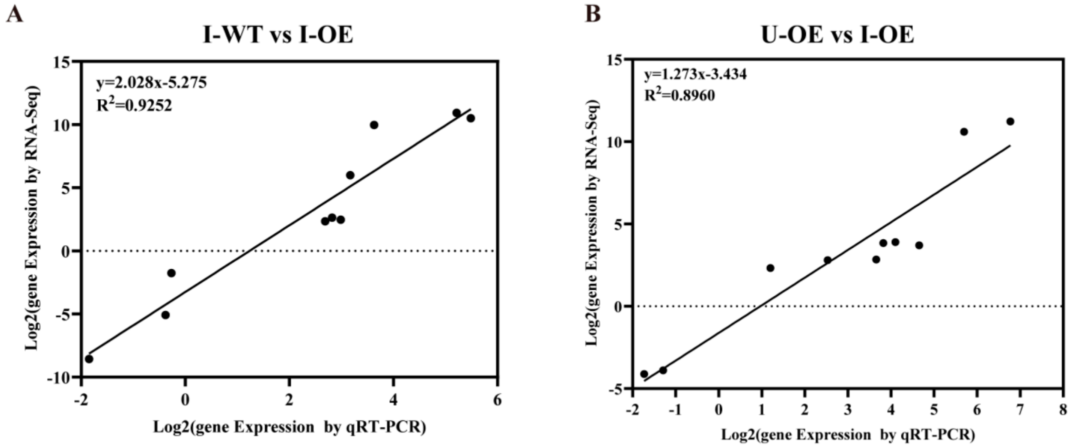

Figure S6. Validation of RNA-seq results by qRT-PCR. A: Linear regression analysis between qRT-PCR and RNA-seq log_2 fold-change values for 10 selected DEGs in I-WT vs. I-OE. B: Linear regression analysis between qRT-PCR and RNA-seq log_2 fold-change values for 10 selected DEGs in U-OE vs. I-OE. Each point represents one validated DEG. The R^2 values indicate the consistency between qRT-PCR and RNA-seq data. Standard locus IDs and primers of the validated genes are listed in Table S1.

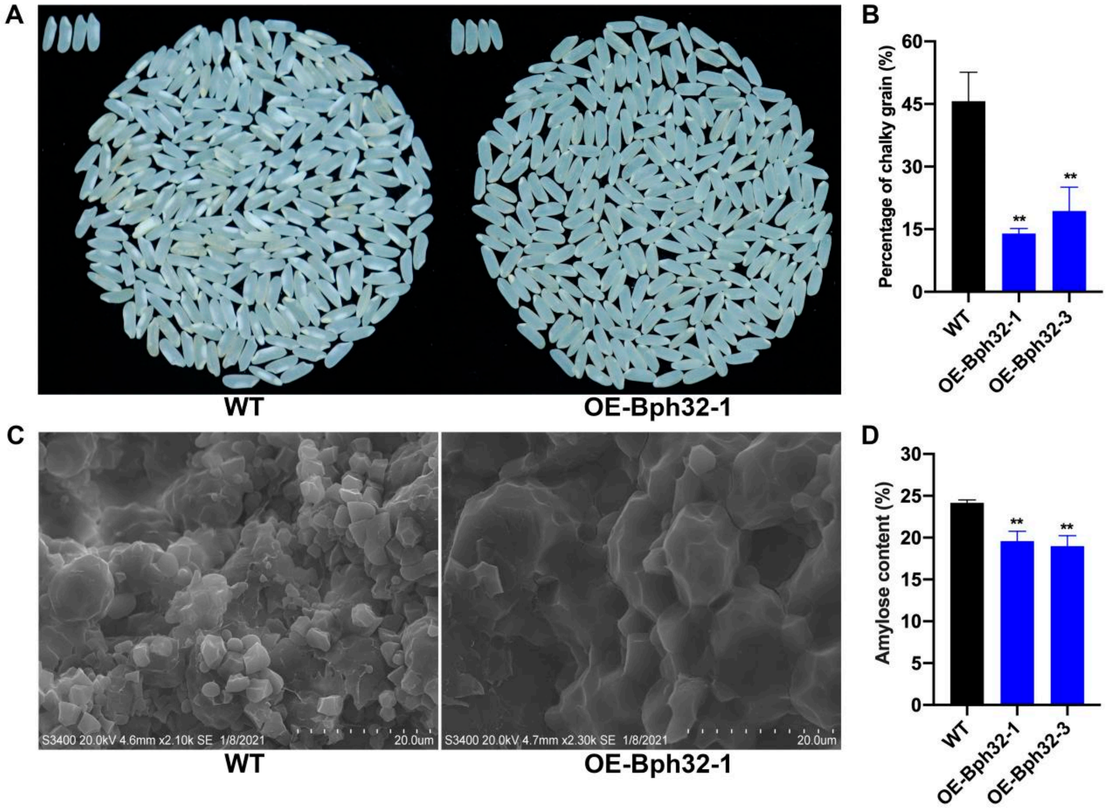

Figure S7. Effect of *OsBph32* overexpression on grain quality. A: Appearance quality of milled rice. B: Scanning electron microscopy observation of cross section of milled rice. Scale bars = 20 μm. C: Chalkiness percentage of milled rice. D: Amylose content of milled rice. Statistical significance was analyzed by Student's t-test. Asterisks indicate significant differences between OE-*Bph32* and WT plants: **, P < 0.01.

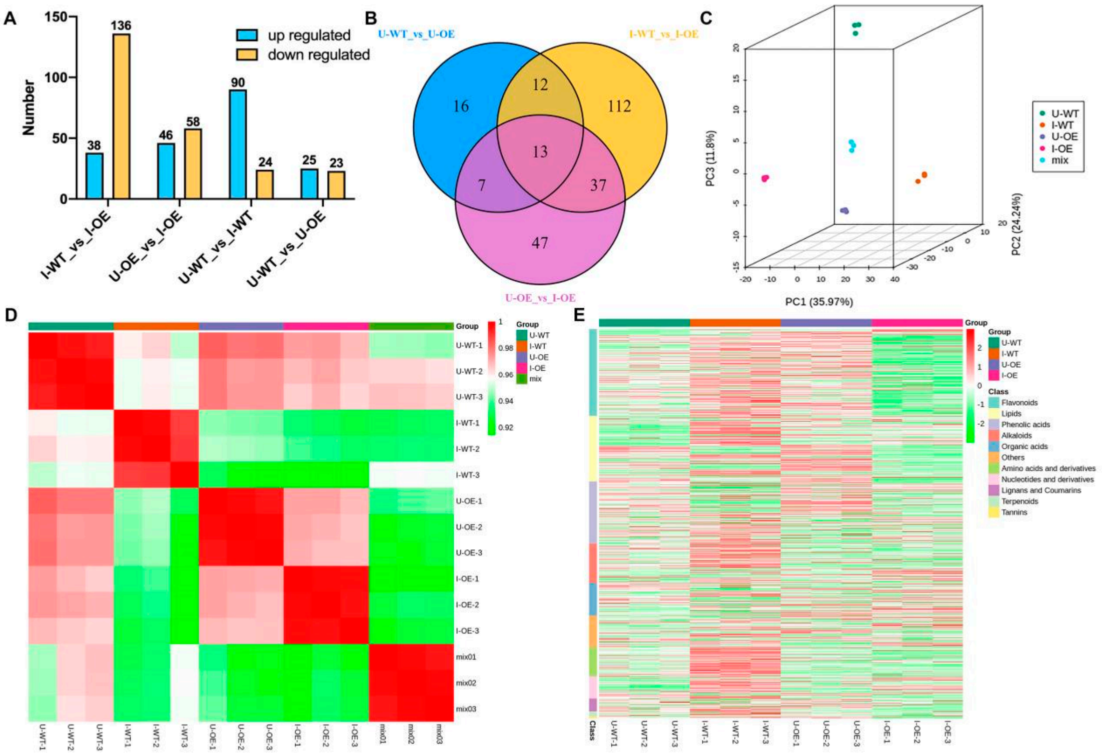

Figure S8. Evaluation of metabolome data. A: Number of differential abundance metabolites (DAMs). B: Venn diagram of DAMs. C: Principal component analysis of the four sample groups. D: Correlation evaluation of the four sample groups. E: Cluster analysis of DAMs in the four sample groups.

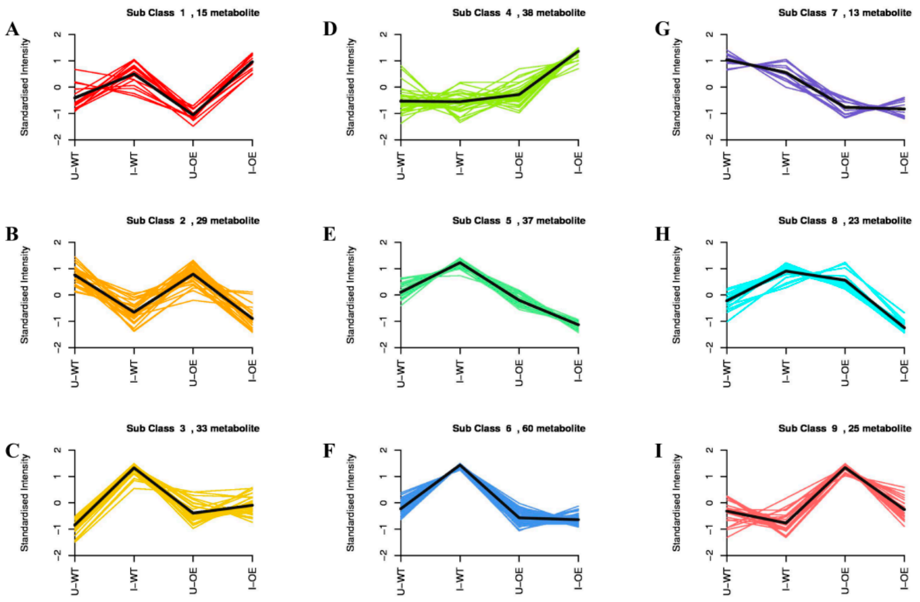

Figure S9. K-means clustering of differentially accumulated metabolites across the four sample groups.

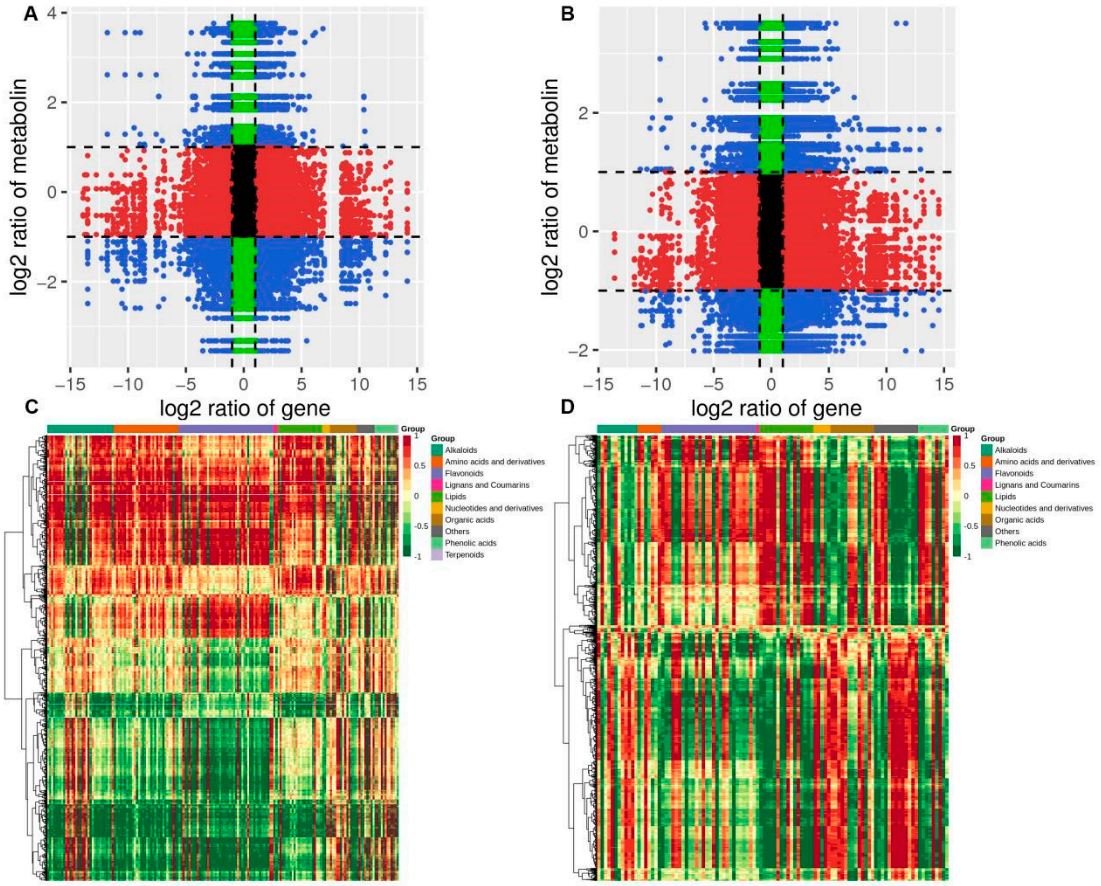

Figure S10. Integrated transcriptome–metabolome correlation analysis. A: Nine-quadrant analysis of gene–metabolite correlations in I-WT vs. I-OE. B: Nine-quadrant analysis of gene–metabolite correlations in U-OE vs. I-OE. C: Correlation coefficient clustering heatmap of gene–metabolite pairs in I-WT vs. I-OE. D: Correlation coefficient clustering heatmap of gene–metabolite pairs in U-OE vs. I-OE. In panels A and B, each point represents a gene–metabolite pair. The x-axis indicates the $\log_2$ fold change of genes, and the y-axis indicates the $\log_2$ fold change of metabolites. Gene–metabolite pairs in different quadrants represent different correlation patterns between transcript and metabolite changes. Panels C and D show the correlation coefficients between differentially expressed genes and differential abundance metabolites, with metabolite classes indicated by different colors.

Table S1. Primers used in this study.

Primers	Forward primer	Reverse primer	Purpose
OE-*Bph32*	GGGGACAAGTTTGTACAAAAAAGCAGGCTTCATGGCAGCGATGATCGGGAC	GGGGACCACTTTGTACAAGAAAGCTGGGTCATAGGTACAGACGTCGTTGATGGAGTA	Vector construction
Hyr	CTGCTGCTCCATACAAGCCAACC	ACATTGGGGAGTTCAGCGAGAGCCT	Transgenic plant screening
35S-*Bph32*	CCATTGCCCAGCTATCTGTCA	GTGACGTTGATCTGCCGCTC	Transgenic plant screening
OsActin	CCTCTTCCAGCCTTCCTTCATAG	CGATGTTGCCATATAGATCCTTCC	Internal reference gene for qRT-PCR
Bph32-qRT	TGGGTTCCGGTGGACCTGGG	GGACGTTGACCCTCGCCGTG	OsBph32 expression validation for Figure A1
GNS5-qRT	ATTGGTCCTTGGAGTTGCG	CGATGCCGTTGGACTTGTA	Callose-related downstream qRT-PCR analysis for Figure A1
GNS6-qRT	ATTGCTCCTTGCAGCATTTC	CTGGACGTTCTGCTTGACC	Callose-related downstream qRT-PCR analysis for Figure A1
GNS9-qRT	TTGCCGCTGCTCTTCCTGT	ACCTTGATGCCGGTGTTGG	Callose-related downstream qRT-PCR analysis for Figure A1
LOC_Os04g43200	TGGCGACGGTATCGTTTATC	CCGTGCTTAGCCCTGTGGA	RNA-seq validation for Figure A6, I-WT vs. I-OE
LOC_Os08g04540	CAACCTGCCGACGAGCTTCATG	CCTTGAAGAACGTGGAGTGCGTCT	RNA-seq validation for Figure A6, I-WT vs. I-OE
LOC_Os05g48700	GTTCTTCAAGGCGACCAACCACG	GTTCGACCCGATGCTCTTGCTG	RNA-seq validation for Figure A6, I-WT vs. I-OE
LOC_Os11g47530	GGCTGTTCACGCACATCCACG	CGGACGTGGATGTGCGTGAA	RNA-seq validation for Figure A6, I-WT vs. I-OE
LOC_Os08g15292	GTAGGTTTAGCGGTTCTCG	TCGGCACAGAAACCATTA	RNA-seq validation for Figure A6, I-WT vs. I-OE
LOC_Os03g08620	AAGCAGAAGAGGCGTAGGAT	CACGGGTTGCCAGGTAGA	RNA-seq validation for Figure A6, I-WT vs. I-OE
LOC_Os05g51820	CTCACGCCCGCATTCTACATC	AGCAGGAGGTGATGAGGGAGC	RNA-seq validation for Figure A6, I-WT vs. I-OE
LOC_Os05g07120	GATACTCCCTGATGCTAAGCAGATGG	ATTGGACGGTCACCTCGTTGG	RNA-seq validation for Figure A6, I-WT vs. I-OE
LOC_Os08g15296	TGGACCAAGACAAACTCG	GAATACCGCGAATAAAGC	RNA-seq validation for Figure A6, I-WT vs. I-OE

LOC_Os01g54420	TCGCCGACCTCA TCAAGCA	GCCAGCGACTTCACC ATT	RNA-seq validation for Figure A6, I-WT vs. I-OE
LOC_Os03g07140	CGAGAAGATAT TGCGGACGAA	CATCTCCGACTACAG GCACCAG	RNA-seq validation for Figure A6, U-OE vs. I-OE
LOC_Os02g40784	ACATCGCCTTCT CCCTCA	CCAACCCATTGAACA GGA	RNA-seq validation for Figure A6, U-OE vs. I-OE
LOC_Os01g61610	TCGTGAACGCCG AGGAGGA	ACACCCTGTACTCGT CGAAGGTCAT	RNA-seq validation for Figure A6, U-OE vs. I-OE
LOC_Os11g47510	CGCCGTCTTCTT CTTCCTCCTC	CGAAGACGCTGAGG AAGGAGATG	RNA-seq validation for Figure A6, U-OE vs. I-OE
LOC_Os01g52240	CGCCAATGCCA AGGTGTTCG	ACCTCCAGCTCCCGG TTCTTGG	RNA-seq validation for Figure A6, U-OE vs. I-OE
LOC_Os02g24642	CGCCAAATTGCC CGAAGC	GCCAAACAAAGGCT AATAGAAA	RNA-seq validation for Figure A6, U-OE vs. I-OE
LOC_Os05g39720	CCTCCTCACGCC CAGTTTATTCC	GGCAGGAACGTGAA GTCCGAGTA	RNA-seq validation for Figure A6, U-OE vs. I-OE
LOC_Os03g08320	ATGAAGGAGCA CAGTGGAAGCA A	TGCGTGTCTTTCAGC GTCCC	RNA-seq validation for Figure A6, U-OE vs. I-OE
LOC_Os03g43860	TCAGGACCAGC AAGAGGG	TTTGGTCGCAGCCTC AT	RNA-seq validation for Figure A6, U-OE vs. I-OE
LOC_Os08g41440	TGCTGTCCCACT CCTTCCACCTC	GCTCCTCCTCACCTC CTTCTCCCAC	RNA-seq validation for Figure A6, U-OE vs. I-OE

Table S2. Statistics of DEGs.

DEG Set	DEG Number	up-regulated	Down-regulated
U-WT vs. U-OE	1723	958	765
I-WT vs. I-OE	2762	1515	1247
U-OE vs. I-OE	6296	3523	2773
U-WT vs. I-WT	3854	2469	1358

Table S3. List of DAMs (Fold change>5).

	Compounds	Class II	VIP	Fold_Change	Log-FC
I-WT vs. I-OE	Genkwanin (Apigenin 7-methyl ether)	Flavonoid	1.23	13.58	3.76
	D-Galactose*	Saccharides and Alcohols	1.20	12.5	3.64
	p-Coumaroyltyramine	Phenolamine	1.23	11.75	3.56
	Acacetin	Flavonoid	1.23	11.28	3.50
	5,4'-Dihydroxy-7-methoxyflavanone (Sakuranetin)	Flavonoid	1.23	10.14	3.34
	Naringenin (5,7,4'-Trihydroxyflavanone)	Dihydroflavone	1.23	8.45	3.08
	D-Mannose*	Saccharides and Alcohols	1.19	7.26	2.86
	Aromadendrin (Dihydrokaempferol)	Dihydroflavonol	1.23	6.96	2.80
	Cinnamoyltyramine	Alkaloids	1.23	6.12	2.61
	2'-Deoxyinosine-5'-monophosphate	Nucleotides and derivatives	1.23	5.95	2.57
U-OE vs. I-OE	Quillaic acid	Phenolic acids	1.28	11.39	3.51
	Naringenin (5,7,4'-Trihydroxyflavanone)	Dihydroflavone	1.28	10.79	3.43
	5,4'-Dihydroxy-7-methoxyflavanone (Sakuranetin)	Flavonoid	1.28	9.18	3.20
	Glutaric acid	Organic acids	1.28	8.45	3.08
	p-Coumaroyltyramine	Phenolamine	1.28	7.52	2.91
	D-Melezitose	Saccharides and Alcohols	1.21	5.59	2.48
	Acacetin	Flavonoid	1.27	5.28	2.40
	Genkwanin (Apigenin 7-methyl ether)	Flavonoid	1.27	5.16	2.37
	Tryptamine	Plumerane	1.28	5.14	2.36
